# Severe, primary, and incidental COVID-19 in hospitalised children, South Africa: 2020–2023

**DOI:** 10.7189/jogh.16.04009

**Published:** 2026-01-30

**Authors:** Ameena Goga, Trisha Ramraj, Jeané Cloete, Dini Mawela, Zainab Waggie, Moherndran Archary, Kogielambal Chinniah, Prakash Jeena, Nomakhuwa E Tabane, Riana Van Zyl, Gary Reubenson, Renate Strehlau, Ute Feucht, Tarylee Reddy, Nobuhle Mchunu, Shannon Cawood, Liesl Zühlke, Kate Webb, Heather J Zar, Kirsten A Donald, Christiaan Scott, Brenda M Morrow, Thomas Aldersley, Nicolette M du Plessis, Terusha Chetty, Sithembiso Velaphi, Ziyaad Dangor, David P Moore

**Affiliations:** 1 South African Medical Research Council (SAMRC) HIV and other Infectious Diseases Research Unit, Durban, South Africa (SA); 2 Department of Paediatrics and Child Health, University of Pretoria, Pretoria, SA; 3 Research Centre for Maternal, Fetal, Newborn and Child Health Care Strategies, University of Pretoria, Pretoria, SA; 4 SAMRC, Maternal and Infant Health Care Strategies Research Unit, Pretoria, SA; 5 Sefako Makgatho Health Sciences, University Department of Paediatrics and Child Health, Pretoria, SA; 6 Department of Paediatrics and Child Health, Chris Hani Baragwanath Academic Hospital, School of Clinical Medicine, Faculty of Health Sciences, University of Witwatersrand, Johannesburg, SA; 7 Department of Paediatrics & Child Health, University of KwaZulu Natal, Durban, SA; 8 Department of Paediatrics and Child Health, Universitas Academic Hospital, University of the Free State, Bloemfontein, SA; 9 Department of Paediatrics & Child Health, Rahima Moosa Mother & Child Hospital, School of Clinical Medicine, Faculty of Health Sciences, University of the Witwatersrand, Johannesburg, SA; 10 SAMRC, Biostatistics Research Unit, Durban, SA; 11 Nelson Mandela Children’s Hospital, University of Witwatersrand, Johannesburg, SA; 12 SAMRC, Office of the Vice President, Cape Town, SA; 13 Division of Paediatric Cardiology, Department of Paediatrics, Red Cross War Memorial Children’s Hospital, University of Cape Town, Cape Town, SA; 14 Department of Paediatric Rheumatology, Red Cross War Memorial Children’s Hospital, University of Cape Town, Cape Town, SA; 15 Department of Paediatrics and Child Health, University of Cape Town, Cape Town, SA; 16 SAMRC Unit on Child & Adolescent Health, University of Cape Town, Cape Town, SA; 17 Department of Paediatrics and Child Health, and Neuroscience Institute, University of Cape Town, Cape Town, SA; 18 Department of Pediatrics, University of Ottawa, Ottawa, Ontario, Canada; 19 Discipline of Public Health Medicine, School of Nursing and Public Health,University of KwaZulu-Natal, Durban, SA; 20 SAMRC Vaccines and Infectious Diseases Analytics Research Unit, Faculty of Health Science, University of the Witwatersrand, Johannesburg, SA

## Abstract

**Background:**

Knowledge gaps persist regarding paediatric COVID-19 clinical presentation, treatment and outcomes in high HIV prevalence settings, with low COVID-19 vaccine coverage.

**Methods:**

An ambi-directional cohort study was conducted in 13 South African public sector hospitals. Hospitalised children with SARS-CoV-2 infection or post-infection syndrome were included. Main outcomes measures included severe disease and primary COVID-19 (hospitalisation for SARS-CoV-2 infection).

**Results:**

There were 2363 SARS-CoV-2 positive children included (March 2020 through May 2023); median age 23.6 months (interquartile range (IQR) = 4.3–98.2 months). Excluding missing values, 1618 (68.9%) children had primary COVID-19; 1121 (69.3%) of these had severe primary COVID-19. In the primary COVID-19 group with data, 318 / 1588 (20.0%) received intensive or high care, 121/1285 (9.4%) received a blood transfusion and 48/1616 (3.0%) died. Multivariable analyses demonstrated that severe primary COVID-19 was 32% higher in children aged 29–365 days (adjusted Risk Ratio (aRR) = 1.32 (95% confidence interval (CI) = 1.13–1.55); reference: 0–28 days), 13% higher with one or more comorbidities (aRR = 1.13; CI = 1.05-1.22)), and 14–22% lower during the Beta, Delta and Omicron periods (reference: ancestral period). Amongst all hospitalised children with a positive SARS-CoV-2 test, severe disease was commoner in underweight children (aRR 1.09; CI = 1.02–1.17, *P* = 0.013)). Severe signs were commoner in children living with HIV (CLHIV), 88/121 (72.7%), *vs*. HIV uninfected 1320 / 2104 (62.7%), *P* = 0.026, and in antiretroviral therapy-naïve CLHIV, (37 / 41 (90.2%), *vs*. CLHIV on therapy 51 / 80 (63.8%), *P* = 0.002).

**Conclusions:**

In a high HIV prevalence country, approximately 70% of children with a positive SARS-CoV-2 test were hospitalised for COVID-19 treatment; almost 70% of these children were severely ill. Controlling for other factors, disease severity was highest in the hypothesised pre-immunity Ancestral period. HIV infection and delayed ART initiation were associated with severe signs. In such settings, strengthening general child health programmes to reduce underweight and prevent or treat paediatric HIV may reduce the severity of new diseases of pandemic proportion.

Coronavirus disease 2019 (COVID-19) following Severe Acute Respiratory Syndrome Coronavirus 2 (SARS-CoV-2) infection caused a 41-month global public health emergency (January 2020–May 2023). Although the pandemic has subsided, there is ongoing concern about emerging viral variants [1]. Furthermore, knowledge gaps persist regarding paediatric COVID-19 clinical presentation, treatment and outcomes in high HIV prevalence settings, with low COVID-19 vaccine coverage, and to our knowledge, no published paediatric study has differentiated between primary *vs*. incidental COVID-19 hospitalisation across COVID-19 variant periods. This provided the scientific rationale for our analysis.

Early data, mainly from high-income settings, reported that 90% children had asymptomatic, mild or moderate disease [2]. Notwithstanding this, some hospitalised children with COVID-19 required respiratory support; furthermore, some children with asymptomatic SARS-CoV-2 infection developed a severe, post-infectious multi-system inflammatory syndrome (MIS-C) [3].

A large multi-country analysis of 31 785 mostly (COVID-19) unvaccinated hospitalised children (Australia (n = 433), Brazil (n = 224), Europe (Italy and Portugal n = 144), South Africa (n = 21 627), Switzerland (n = 969), Thailand (n = 67), UK (n = 8127) and the USA (n = 194), March 2020–March 2022), demonstrated more intensive care unit (ICU) admissions, ventilatory support and oxygen use in children aged 5–18 years during the Ancestral wave, with improvement in all three outcomes as the pandemic progressed [4]; however, in children <5 years, only ICU admission improved over the same period [4]. No data were reported on clinical care, laboratory indices and clinical management, and there was no separate analysis of outcomes in the 449 HIV exposed/positive children. An earlier retrospective analysis (March–December 2020), of Ancestral wave data from 469 COVID-19 unvaccinated hospitalised children with PCR-confirmed SARS-CoV-2 infection in sub-Saharan Africa (SSA) – Democratic Republic of the Congo, Ghana, Kenya, Nigeria, South Africa, and Uganda – reported high rates of ICU admission (35.0%) and death (8.3%) with notable regional differences [5]. No data were reported on subsequent COVID-19 waves, and the inclusion of only 11 (2.3%) children living with HIV (CLHIV), precluding in-depth analyses in this group.

In India and Pakistan, data from five tertiary hospitals per country (January 2020–March 2022 and January 2020–December 2021, respectively) described severe COVID-19 in 65.0 and 71.4% of COVID-19 unvaccinated hospitalised children (n = 2148 and n = 1090 respectively) [6,7]. Mortality rates were 13.3 and 18.6%, respectively. Co-morbidity analysed did not include HIV or tuberculosis (TB). In India, data were presented by three surges – predominantly Ancestral, Delta and Omicron variant periods – and demonstrated higher severe acute COVID-19 cases and MIS-C cases during the Ancestral and Delta periods respectively. Mortality risk was highest for MIS-C during the Ancestral period (26%), followed by severe acute COVID-19 during the Delta period (22%). In Pakistan, analysis of COVID-19 severity and outcome by variant were not reported. In South Africa, where COVID-19 vaccination for children over 12 years commenced on the 10th October 2021 [8] and opened to high-risk children aged 5–11 years in March 2023, initial data from the National Health Laboratory Service (January–September 2020) described that children <18 years accounted for 2.9% of COVID-19 hospitalisations, but this increased to 5.8% by February 2022 [9,10]. Overall, there was increased paediatric testing and case rates during the Delta and Omicron periods, and comorbidities increased the odds of severe disease in children.

Our multi-centre cohort addressed a gap not covered by national surveillance: we described the clinical presentation, severity, treatment and outcome of COVID-19 amongst SARS-CoV-positive, hospitalised children, across four SARS-CoV-2 variant periods, and the association between HIV-infection and SARS-CoV-2 infection in South Africa, March 2020–May 2023. Furthermore, based on an adjudication process, we differentiate between children hospitalised for SARS-CoV-2 infection (primary COVID-19) and children hospitalised for other illnesses, where the routine SARS-CoV-2 test was positive (incidental COVID-19). Data analysis is presented in **Figure 1** and in the **Online Supplementary Document**.

**Figure 1:**
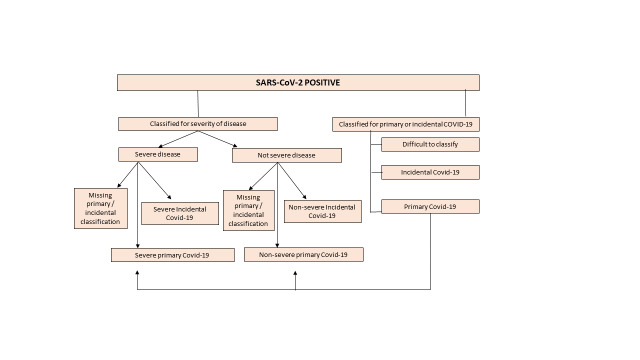
Flowchart. Detailed description is provided in the first section of the **Online Supplementary Document**.

We did not include follow-up data post-discharge. This study was part of a World Health Organization (WHO)-coordinated multi-country (Ethiopia, India, Pakistan and South Africa) study on severe COVID-19 in hospitalised children.

## METHODS

### Study design

Data were gathered from parental interviews (if parents were contactable), and medical records, during (prospective cohort) or after (retrospective cohort) hospitalisation.

### Study population

Children were recruited from 13 public hospitals, purposively selected to ensure geographical spread across four of the nine provinces in South Africa, and where clinicians were willing to contribute towards a paediatric COVID-19 study (Table S1 in the **Online Supplementary Document**). Children aged 0–19 years were eligible if they presented with any new illness from 1 January 2020 and had a positive SARS-CoV-2 real-time PCR or rapid antigen test. Children with positive SARS-CoV-2 serology and/or MIS-C were also eligible for inclusion [11]. During the study period, SARS-CoV-2 PCR testing was available from 3 March 2020 (Table S1 and Text Box 1 in the **Online Supplementary Document**). Antibody and antigen tests were authorised for use in South Africa from 25 August 2020 and 1 October 2020, respectively [12,13], however, their availability was limited.

### Data collection

Trained study staff, including paediatricians, nurses, and data entry clerks collected data. To ensure consistent data abstraction and interviewing across all sites, the South African Medical Research Council (SAMRC) trained study staff trained using a study-specific standardised operating procedure (SOP). Data were recorded on paper forms and subsequently entered into the Research Electronic Data capture (REDCap) database [14,15] hosted at the SAMRC, within two days. The SAMRC conducted weekly meetings to monitor study progress, ensure data quality, consistency and identify and mitigate barriers to data collection. A quality control system was established at each site and overseen by the SAMRC. Prospective data collection started on 21 June 2021. Hospitalisations before this date were captured retrospectively. In the Western Cape and Free State provinces, data collection ended on 31 December 2021. In Gauteng and KwaZulu-Natal provinces, data collection ended on 31 March 2025. The study protocol (for data collection until 31 December 2021) was registered in the ANZ clinical trials registry (ACTRN12621001154897) [16].

### Data analysis

For this analysis, the database was locked on 1 June 2023, three weeks after WHO declared an end to the COVID-19 pandemic [1].

We defined SARS-CoV-2 exposure, acute COVID-19, severe disease, SARS-CoV-2-related disease, MIS-C, and undernutrition as per study protocol (**Figure 1**), and similar to the definitions used in papers published by Gupta et al. and Abbas et al. [6,7]. During data analysis we defined primary COVID-19, severe primary COVID-19, incidental COVID-19, and COVID-19 variant periods (Figure S1 in the **Online Supplementary Document****)** [17]. Definitions for incidental COVID-19 were guided by Klann et al. [18], with adaptations for a paediatric population. Severe disease was defined as

(i) age-specific respiratory distress and/or hypotension, or

(ii) hypoxaemia and/ or requiring supplemental oxygen, vasopressors, non-invasive or mechanical ventilation, or

(iii) death.

Primary COVID-19 was defined as a hospitalisation primarily because of symptoms relating to SARS-CoV-2 infection (including MIS-C). Severe primary COVID-19 was defined as a severe disease in a child with primary COVID-19.

The study team designated a new variant period as having started when 50% of specimens collected nationally yielded this new genome [17]. National data were used to identify variant periods because provincial-level variant data were sparse [17]. The dates used to identify variant periods are similar to the dates used for a South Africa cohort (T1, T2 and T3) in a publication by Zhu et al. [4], except that we differentiated between two variants during T2. Individual-level genome testing to identify variants was not undertaken.

We described the characteristics of all enrolled children. During data analysis, an expert group of nine paediatricians adjudicated all cases to determine if they were primary or incidental COVID-19 (**Figure 1**), guided by an SOP (the last section of the **Online Supplementary Document**). SAMRC oversaw this process. Each case was independently adjudicated by two paediatricians blinded to the case’s origin and to each other. A third paediatrician adjudicated discordant decisions, blinded to the other adjudications. Each paediatrician submitted their adjudications before the scheduled consensus meetings where all cases and adjudications were reviewed. Furthermore, the diagnoses of children with incidental COVID-19 were reviewed and the charts of children with any diagnoses that flagged primary COVID-19 *e.g*. respiratory illness were re-reviewed. Any remaining discrepancies were discussed during consensus meetings. Despite these discussions, a subset of cases was difficult to classify.

During data analysis, underweight for age was derived: Fenton growth charts were used in premature/ ex-premature neonates/infants to minimise over-attribution of underweight for age (**Figure 1**). Categorical variables were summarised using frequencies and percentages. The χ^2^ or Fisher exact test were used to test associations between categorical variables. The Shapiro-Wilk test was used to test normality of continuous variables. Normally distributed variables were summarised using means and standard deviations, with differences tested using ANOVA or the unpaired *t* test. Skewed variables were summarised using medians and interquartile ranges (IQR), and differences testing using the Kruskal-Wallis or Wilcoxon rank sum test. Factors associated with severe primary COVID-19, and severe disease were analysed using Poisson regression with robust standard errors. This approach is preferred when the outcome is common, as the odds ratio from logistic regression can substantially overestimate the true risk ratio. In addition, this approach provides consistent estimates of the risk ratio and is computationally stable, even in situations where alternative models, such as log-binomial regression, may fail to converge. All analysis was performed using Stata 17.0 (StataCorp, College Station, TX, USA).

## RESULTS

We enrolled 2363 children with SARS-CoV-2, across four COVID-19 variant periods: 350 (14.8%) during the 8-month Ancestral period, 550 (23.3%) during the 7-month Beta period, 603 (25.5%) during the 5-month Delta period and 860 (36.4%) during the 19-month Omicron period. Vaccination uptake was very low (0.5%) (**Table 1**; Figure S1 in the **Online Supplementary Document**).

**Table 1:** Characteristics of all SARS-CoV-2-positive children by variant period*

	Ancestral, n = 350 (14.8%)	Beta, n = 550 (23.3%)	Delta, n = 603 (25.5%)	Omicron, n = 860 (36.4%)	Total, n = 2363 (100%)	*P*-value	Missing
	**March 2020 – 4 November 2020**	**5 November 2020 – 2 June 2021**	**3 June 2021 -10 November 2021**	**11 November 2021 -31 May 2023**			
**Province**						<0.001	0
Free State	20 (5.7)	37 (6.7)	36 (6.0)	0 (0.0)	93 (3.9)		
Gauteng	130 (37.1)	249 (45.3)	372 (61.7)	713 (82.9)	1464 (62.0)		
KwaZulu-Natal	34 (9.7)	133 (24.2)	67 (11.1)	126 (14.7)	360 (15.2)		
Western Cape	166 (47.4)	131 (23.8)	128 (21.2)	21 (2.4)	446 (18.9)		
**Data collection method**						<0.001	446
Retrospective	181 (98.4)	414 (98.8)	323 (68.0)	239 (28.5)	1157 (60.4)		
Prospective	3 (1.6)	5 (1.2)	152 (32.0)	600 (71.5)	760 (39.6)		
Age in months (MD IQR)	28.9 (4.4–108.2)	26.9 (3.5–121.3)	26.9 (4.5–112.8)	19.2 (4.5–69.2)	23.6 (4.3–98.2)	0.005	5
**Age category**						<0.001	35
0–28 d	44 (12.7)	49 (9.1)	53 (9.0)	64 (7.5)	210 (9.0)		
29–365 d	75 (21.6)	152 (28.3)	163 (27.6)	290 (34.0)	680 (29.2)		
1–5 y	99 (28.5)	117 (21.7)	136 (23.1)	258 (30.2)	610 (26.2)		
5–12 y	78 (22.5)	113 (21.0)	150 (25.4)	184 (21.6)	525 (22.6)		
>12 y	51 (14.7)	107 (19.9)	88 (14.9)	57 (6.7)	303 (13.0)		
**Nutritional status**						0.053	335
Normal/overweight	241 (78.8)	301 (71.5)	383 (74.1)	556 (70.9)	1481 (73.0)		
Underweight†	65 (21.2)	120 (28.5)	134 (25.9)	228 (29.1)	547 (27.0)		
Severe disease	232 (66.3)	359 (65.3)	364 (60.4)	545 (63.4)	1500 (63.5)	0.21	0
**Type of COVID-19**						<0.001	14
Incidental	64 (18.8)	183 (33.5)	195 (32.4)	237 (27.6)	679 (28.9)		
Primary	275 (80.6)	357 (65.4)	399 (66.3)	587 (68.3)	1618 (68.9)		
Difficult to adjudicate	2 (0.6)	6 (1.1)	8 (1.3)	36 (4.2)	52 (2.2%)		
**Child’s HIV status**						0.47	139
Positive	14 (4.2)	24 (4.8)	31 (5.4)	52 (6.3)	121 (5.4)		
Negative	320 (95.8)	471 (95.2)	539 (94.6)	773 (93.7)	2103 (94.6)		
**History of COVID-19 in the household**						<0.001	438
Yes	34 (11.6)	38 (10.1)	55 (12.1)	36 (4.5)	163 (8.5)		
No	258 (88.4)	339 (89.9)	398 (87.9)	767 (95.5)	1762 (91.5)		
**SARS-CoV-2 RT-PCR result**						<0.001	0
Positive	348 (99.4)	547 (99.5)	597 (99.0)	829 (96.4)	2321 (98.2)		
Negative	2 (0.6)	1 (0.2)	2 (0.3)	1 (0.1)	6 (0.3)		
Not done	0 (0.0)	2 (0.4)	4 (0.7)	30 (3.5)	36 (1.5)		
**SARS-CoV-2 Rapid antigen test**						<0.001	0
Positive	0 (0.0)	12 (2.2)	6 (1.0)	34 (4.0)	52 (2.2)		
Negative	1 (0.3)	2 (0.4)	7 (1.2)	2 (0.2)	12 (0.5)		
Not done	349 (99.7)	536 (97.5)	590 (97.8)	824 (95.8)	2299 (97.3)		
**SARS-CoV-2 Rapid antibody or ELISA test**						0.002	1
Positive	3 (0.9)	3 (0.5)	6 (1.0)	8 (0.9)	20 (0.8)		
Negative	5 (1.4)	11 (2.0)	14 (2.3)	46 (5.4)	76 (3.2)		
Not done	342 (97.7)	536 (97.5)	583 (96.7)	805 (93.7)	2266 (95.9)		
**Child received a COVID-19 vaccine?‡**						0.37	874
Yes	0 (0.0)	0 (0.0)	2 (0.6)	6 (0.8)	8 (0.5)		
No	112 (100.0)	281 (100.0)	352 (99.4)	736 (99.2)	1481 (99.5)		
**Mother received a COVID-19 vaccine‡**						<0.001	1200
Yes	17 (23.9)	23 (12.9)	54 (19.9)	232 (36.1)	326 (28.0)		
No	54 (76.1)	155 (87.1)	217 (80.1)	411 (63.9)	837 (72.0)		
**Father received a COVID-19 vaccine†**						<0.001	1381
Yes	15 (26.3)	24 (14.6)	38 (16.1)	163 (31.0)	240 (24.4)		
No	42 (73.7)	140 (85.4)	198 (83.9)	362 (69.0)	742 (75.6)		

*Presented as n (%) unless specified otherwise.

†Underweight: Weight for age less than -2 Z score using Fenton and WHO growth charts.

‡COVID-19 vaccination in South Africa commenced on 18 May 2021 for adults, and on 10 October 2021 for children aged 12 y and above.

### Description of overall study population

Most (60.4%) children were enrolled retrospectively (after hospital discharge) (**Table 1**). Median age was 23.6 months, overall and 27.0% were underweight-for-age (UFA) at admission (**Table 1**) with significantly younger age and higher UFA as waves progressed (*P* = 0.005). There were 121 (5.4%) CLHIV which did not vary significantly by wave (Table S2 in the **Online Supplementary Document**). Similarly, there was no association between TB and COVID-19 variant periods (Table S2 in the **Online Supplementary Document**). There was a significant increase in incidental COVID-19 and a decrease in primary COVID-19 between the Ancestral and other waves (**Table 1**).

### Severity of disease, and symptoms across variant periods (overall population)

Amongst all participants, 63.5% were classified as having severe disease with no difference between the variant periods, *P* = 0.21 (**Table 1**); 3.2% died (Table S3 in the **Online Supplementary Document**). Supplementary symptoms varied significantly through the variant periods: fever, wheezing, abdominal pain, skin rash/peeling skin, joint swelling/pain, muscle pain, tachycardia, hypotension, prolonged capillary refill time, and pale/mottled skin were commoner during the Ancestral period (Table S2 in the **Online Supplementary Document**).

### Treatment and outcomes across all four variant periods (overall population)

There was no significant difference in the need for oxygen supplementation across the four periods (34.7%, *P* = 0.16; Table S3 in the **Online Supplementary Document**); however, the Omicron period was associated with a shorter median duration of oxygen therapy (3 days, IQR = 2–5 days *vs*. 5 days, IQR = 2–8 days for the Ancestral period, *P* = 0.009); there were fewer blood transfusions (7.4% in the Omicron period *vs*. 11.3, 12.6, and 11.4% in the Ancestral, Beta and Delta periods respectively, *P* < 0.001), and fewer deaths (2.7% *vs*. 5.7% during the Ancestral period, *P* < 0.001). Supplementary tables describe the study population by variant period (Table S2–3 in the **Online Supplementary Document**), and by primary and incidental COVID-19 (Table S4 in the **Online Supplementary Document**).

### Risk factors for severe disease, and association between HIV and COVID-19 (overall population)

In unadjusted univariate analysis, signs of severe disease occurred more frequently in CLHIV (n/N = 88/121, 72.7%) than children without HIV (n/N = 1320/2104, 62.7%, *P* = 0.026) (Table S5 in the **Online Supplementary Document**). Amongst CLHIV, those not on ART were more likely to have severe disease (n/N = 37/41, 90.2%) compared with those on ART (n/N = 51/80; 63.8%, *P* = 0.002). Compared with CLHIV on ART, there was a tendency for more ART-naïve CLHIV to require oxygen (n/N = 19/41, 46.3% *vs*. n/N = 22/68, 32.4%, *P* = 0.158) and invasive or non-invasive ventilation (n/N = 8/40, 20% *vs*. 6/70, 8.6%, *P* = 0.134, respectively) (Table S2 in the **Online Supplementary Document**). In multivariable analysis the risk of severe disease (n = 1500), increased if the child was underweight (aRR = 1.09; 95% CI = 1.02–1.17, *P* = 0.013), age 29–365 days or 1–5 years or >12 years (aRR = 1.49; 95% CI = 1.30–1.72, *P* < 0.001), aRR = 1.26; 95% CI = 1.09–1.4, *P* = 0.02; and aRR = 1.51; 95% CI = 1.30–1.77, *P* < 0.001, respectively) or had any comorbidity (aRR = 1.14; 95% CI = 1.07–1.22, *P* < 0.001), and decreased during the Delta period (aRR = 0.89; 95% CI = 0.81–0.99, *P* = 0.026) (Table S9 in the **Online Supplementary Document**).

### Primary *vs*. incidental COVID-19

Excluding those with missing values (n = 14), 1618 (68.9%) of hospitalised children had primary COVID-19 (**Table 1**, **Table 2**; Table S4–8 in the **Online Supplementary Document**). The prevalence of underweight for age was 28.8% in the primary COVID-19 group with significant increased prevalence as the waves progressed in the primary COVID-19 group (*P* = 0.009) (Table S4 in the **Online Supplementary Document**). Amongst children with primary COVID-19, 1121 (69.3%) were classified as severe primary COVID-19 (Table S6–7 in the **Online Supplementary Document**) and 1121 / 2363 (47.4%) of the cohort had severe disease as a result of COVID-19 (severe primary COVID-19). In the primary COVID-19 group, 40 (2.6%) had a history of TB; 81 (5.2%) were CLHIV (Table S7 in the **Online Supplementary Document**).

**Table 2:** Investigations, management and outcome in children with primary COVID-19*

	Severe primary COVID-19, (n = 1121)	Non-severe primary COVID-19, (n = 497)	Total (n = 1618)	*P*-value	Missing
**Investigations conducted**					
Bacteraemia detected	110 (17.4)	32 (16.0)	142 (17.0)	0.65	785
Chest x-ray performed	507 (49.6)	86 (18.1)	593 (39.6)	<0.001	121
Computer tomography (CT) scan performed	30 (2.8)	9 (1.9)	39 (2.5)	0.31	68
Echocardiography (ECHO) performed	98 (9.4)	18 (4.1)	116 (7.8)	<0.001	134
Features of myocardial dysfunction on ECHO	19 (30)	4 (31)	23 (30)	0.97	40
Features of pericarditis on ECHO	3 (21)	0 (0)	3 (17)	0.31	98
Features of valvulitis on ECHO	1 (7)	0 (0)	1 (6)	0.63	99
Coronary abnormalities on ECHO	8 (57)	3 (75)	11 (61)	0.52	98
**Therapies received during hospitalisation**					
Antibiotic	911 (83.6)	296 (66.8)	1207 (78.7)	<0.001	85
Intravenous (IV) fluids	550 (52.8)	189 (44.2)	739 (50.3)	0.003	149
Corticosteroids	208 (19.2)	27 (6.1)	235 (15.4)	<0.001	92
IV immune globulin	57 (5.2)	14 (3.1)	71 (4.6)	0.078	83
Antifungal	93 (10.2)	14 (4.0)	107 (8.5)	<0.001	362
Systemic anticoagulation	97 (8.9)	22 (5.0)	119 (7.8)	0.009	85
Oxygen supplementation received	543 (61.8)	0 (0.0)	543 (44.7)	<0.001	403
Number of days of oxygen therapy received amongst those on oxygen, MD (IQR)	4.0 (2.0–6.0)	0	4.0 (2.0–6.0)		93
Inotropes/vasopressors	48 (5.1)	0 (0.0)	48 (3.7)	<0.001	336
Blood transfusion	110 (11.8)	11 (3.2)	121 (9.4)	<0.001	333
Renal replacement therapy or dialysis	11 (1.3)	0 (0.0)	11 (0.9)	0.038	404
Admitted to Intensive care unit (ICU)	87 (18.0)	9 (4.1)	96 (13.6)	<0.001	914
Admitted to ICU or high dependency unit admission?	300 (27.2)	18 (3.7)	318 (20.0)	<0.001	30
Number of days in ICU, if admitted to ICU	5.0 (4.0–7.0)	4.0 (1.0–5.0)	5.0 (4.0–7.0)	0.042	
Non-invasive ventilation? (*e.g*. BiPAP/CPAP)	85 (9.1)	0 (0.0)	85 (6.6)	<0.001	336
Invasive ventilation	105 (11.2)	0 (0.0)	105 (8.2)	<0.001	330
Duration of invasive ventilation, if received invasive ventilation, MD (IQR)	5.0 (3.0–9.0)		5.0 (3.0–9.0)		21
Outcome: death	48 (4.3)	0 (0.0)	48 (3.0)	<0.001	100

MD – median, IQR – interquartile range

*Presented as n (%) unless specified otherwise.

### Symptoms and comorbidities in children with primary COVID-19

The commonest symptoms and signs significantly more prevalent amongst children with severe primary compared with non-severe primary COVID-19 were difficult or fast breathing (63.9%), cough (55.6%), fever (50.9%), tachypnoea (48%), tachycardia (44.5%), and hypotension (12.6%) (Table S6–7 in the **Online Supplementary Document**), *P* < 0.001. At least one comorbidity was present in 44.9% with severe primary COVID-19, compared with 33.6% in the non-severe primary COVID-19 group (*P* < 0.001), with prematurity (19.4% in the severe primary COVID-19 group *vs*. 10.2% in the non-severe primary COVID-19 group; *P* < 0.001), heart disease (6.2 *vs*. 2.7%, respectively; *P* = 0.004), and chronic lung disease (4.5 *vs*. 2.1%, respectively; *P* = 0.021) being the three commonest comorbid conditions (Table S7 in the **Online Supplementary Document**). There was no difference in the prevalence of TB, HIV infection, diabetes, neurological disease (*e.g*. epilepsy) or asthma amongst children with severe primary COVID-19 and non-severe primary COVID-19 (Table S7 in the **Online Supplementary Document**).

### Investigations, treatment and outcomes amongst children with primary Covid-19

There was no significant difference in detection of bacteraemia between children with severe and non-severe primary COVID-19 (**Table 2**). Significantly more children with severe primary COVID-19 had chest x-rays (49.6 *vs*. 18.1%, *P* < 0.001) and echocardiograms (ECHO) (9.4 *vs*. 4.1%, *P* < 0.001) (**Table 2**). In the group who had an ECHO there was no difference in ECHO findings between the severe and non-severe primary COVID-19 groups (**Table 2**). Amongst children with primary COVID-19, those with severe disease had significantly higher C-reactive protein (18.0; 4.0–68.0) *vs*. 11.0 (95% CI = 2.0–40.0, *P* = 0.002) and D-dimer values (2.1; 95% CI = 0.8–7.5) *vs*. 0.7 (95% CI = 0.3–3.1; *P* < 0.001) and significantly lower lymphocyte counts (3.8; 95% CI = 1.9–7.0) *vs*. 4.8 (95% CI = 2.6–8.2, *P* = 0.002), compared to those with non-severe primary COVID-19 (Table S8 in the **Online Supplementary Document**).

Hospitalised children with severe primary COVID-19 were frequently managed with antibiotics compared with the non-severe group (83.6 *vs*. 66.8%, *P* < 0.001) **(****Table 2****)**. Furthermore, intravenous fluids (52.8 *vs*. 44.2%, *P* = 0.003) and corticosteroids (19.2 *vs*. 6.1%, *P* < 0.001) were more frequently used to treat children with severe primary COVID-19 compared with the non-severe group. Of note is that not all children with severe primary COVID-19 received oxygen therapy or intravenous fluids (**Table 2**). Amongst 1121 children with severe primary COVID-19, 11.8% received a blood transfusion, and 27.2% were admitted to intensive or high care compared with 3.2 and 3.7% in the non-severe primary COVID-19 group; 11.2% received invasive ventilation, and 4.3% died in-hospital (**Table 2**).

### Risk factors for severe primary COVID-19

In multivariable analysis (**Table 3**), the risk of severe primary COVID-19 increased if the child was age 29–365 days (aRR = 1.3; 95% CI = 1.13–1.55) compared with age 0–28 days, or had a comorbidity (aRR = 1.1; 95% CI = 1.05–1.22), and decreased during the Delta (aRR = 0.78; 95% CI = 0.69–0.88) and Omicron (aRR = 0.83; 95% CI = 0.75-0.92) periods (**Table 3**).

**Table 3:** Factors associated with severe primary COVID-19 in hospitalised children with primary COVID-19

	Univariable			Multivariable		
	**Risk ratio**	**95% confidence interval**	***P*-value**	**Risk ratio**	**95% confidence interval**	***P*-value**
**Nutritional status**						
Normal weight	Reference			Reference		
Underweight	1.14	1.06–1.22	<0.001	1.06	0.97–1.15	0.188
Overweight	1.00	0.86–1.17	0.997	0.97	0.80–1.17	0.732
**Age category**						
0–28 d	Reference			Reference		
29–365 d	1.25	1.08–1.43	0.002	1.32	1.13–1.55	0.001
1–5 y	1.02	0.88–1.19	0.793	1.06	0.89–1.26	0.524
5–12 y	1.00	0.85–1.17	0.997	1.03	0.86–1.23	0.761
>12 y	1.25	1.07–1.46	0.004	1.18	0.95–1.46	0.129
**Days since symptom onset**						
<3 d	Reference			Reference		
3–6 d	1.03	0.96–1.11	0.414	1.01	0.93–1.10	0.877
>7 d	1.05	0.96–1.16	0.283	1.02	0.91–1.13	0.792
**Variant**						
Ancestral	Reference			Reference		
Beta	1.01	0.92–1.12	0.772	0.86	0.76–0.97	0.017
Delta	0.94	0.84–1.04	0.202	0.78	0.69–0.88	<0.001
Omicron	0.98	0.89–1.07	0.614	0.83	0.75–0.92	<0.001
**Comorbidities**						
0	Reference			Reference		
1 or more	1.13	1.04–1.21	0.001	1.13	1.05–1.22	0.001

## DISCUSSION

We reported detailed data on clinical presentation, laboratory indices, investigations, treatment and outcomes from one of the largest paediatric cohorts across multiple provinces in a high HIV prevalence LMIC setting, and differentiate between primary *vs*. incidental COVID-19. We highlighted several novel findings: 68.9% of SARS-CoV-2 positive hospitalisations were for primary COVID-19; 69.3% of these were severely ill. Controlling for other factors, clinical signs and outcomes were worst in the Ancestral, (hypothesised) pre-immunity period, necessitating longer oxygen therapy and higher use of intensive care and related interventions *e.g*. inotropes. Though HIV was not independently associated with severity, HIV infection, and lack of ART were associated with worse outcomes. While simple descriptive analyses demonstrate similar prevalence of severe and primary COVID-19 across variant waves, multi-variable analyses demonstrate that severe disease and mortality was higher during the Ancestral period.

Severe primary COVID-19 was significantly associated with age 29–365 days or one or more comorbidities. We describe a trend towards a milder disease phenotype (lower mortality, shorter duration of oxygen therapy, reduced need for ventilation or intensive care) with pandemic progression. These findings are similar earlier South African analyses [9,19] which hypothesised increasing natural and herd immunity against SARS-CoV-2, less effective viral evasion of the interferon response by Omicron, a more prepared or experienced health system, and an increasing level of protection through passively acquired maternal antibodies or vaccination [11]. COVID-19 vaccine coverage amongst children in South Africa was low; hence vaccination does not explain these changes.

Data from high-income countries demonstrate that SARS-CoV-2 infection is generally mild in children [20-23]. Amongst children with a positive SARS-CoV-2 test from 77 institutions in 21 high-income countries (mostly tertiary or quaternary paediatric infectious diseases or paediatric pulmonology units) during the Ancestral period, 13.2%, required ICU admission, and of those hospitalised 1.1% died [20]. In comparison, we found that 22.3% needed ICU or high care in the Ancestral period, declining to 13.4% during the Omicron period, and 3.2% died. Amongst children with primary COVID-19, the proportion who died was almost 3- to 6-fold higher (3.0% overall), than described among European cohorts. Similarly, increased comparative fatality rates have been described in India and Pakistan, with 13.3 and 18.6% mortality, respectively [6,7]. These findings may reflect the existence of different ICU admission thresholds, resource contexts, and may demonstrate that paediatric COVID-19 in African and Asian settings is associated with poorer outcomes.

This disparity in COVID-19 presentation and outcome between African/Asian, and high-income settings may be associated with high rates of malnutrition, background mortality rates, or lower availability of life-saving treatments. We identified malnutrition as a driver of hospitalisation and a potentially modifiable risk factor for severe COVID-19 in children. Just over one-quarter of children hospitalised with COVID-19 were underweight-for-age, 4–5 times higher than the reported prevalence of underweight in South Africa [24] This may reflect the underlying pervasive effect of malnutrition on child health, more than the effect of malnutrition on COVID-19 severity [25].

Our analysis includes 121 CLHIV co-infected with SARS-CoV-2, unlike previous sub-Saharan African data which included only 11 CLHIV [5]. Although HIV was not a significant risk factor for severe disease or severe primary COVID-19, the importance of early ART initiation in high HIV prevalence settings to optimise outcomes is highlighted.

### Limitations

Our study has several limitations including lack of long-term follow-up (especially relevant for MIS-C), misclassification risk due to gaps in symptom documentation, and selection bias due to medical record availability. Furthermore, hospitals were purposively selected; however, these were all public health facilities, representative of paediatric hospitals nationally. Children were mostly recruited retrospectively because COVID-19 hospital policies limited parental access to hospitalised children, increasing reliance on medical record reviews [26]. Furthermore, SARS-CoV-2 genotyping was not routinely undertaken; TB and HIV may be under-reported (albeit only 61 and five were missing, respectively) or misattributed and diagnosis of SARS-CoV-2 infection depended on routine testing policies. Attribution of primary COVID-19 was sometimes difficult, especially in children with comorbidities. However, the same clinicians undertook all adjudications, ensuring consistency. The effect of viral or bacterial co-infection (notably bacteraemia present n 9.7%) on disease severity and long-term outcome data was not analysed. Lastly, we used convenience sampling of as many hospitalised children as possible, thus, there is no population denominator.

Notwithstanding these limitations, this large cohort (n = 2363) covers four geographical areas in a high HIV prevalence country, and includes data across all four variant periods to the end of the pandemic emergency period.

## CONCLUSIONS

We demonstrated that SARS-CoV-2 infection SARS-CoV-2 may lead to severe disease in hospitalised children in high HIV prevalence settings. Our data suggest that strengthening general child health programmes to reduce underweight and prevent or treat paediatric HIV may reduce the severity of new diseases of pandemic proportion, such as COVID-19.

## Additional material


Online Supplementary Document
