# Erratum: Goga A, Ramraj T, Cloete J, Mawela D, Waggie Z, Archary M, Chinniah K, Jeena P, Tabane NE, Van Zyl R, Reubenson G, Strehlau R, Feucht U, Reddy T, Mchunu N, Cawood S, Zühlke L, Webb K, Zar HJ, Donald KA, Scott C, Morrow BM, Aldersley T, du Plessis NM, Chetty T, Velaphi S, Dangor Z, Moore DP. Severe, primary, and incidental COVID-19 in hospitalised children, South Africa: 2020–2023. J Glob Health. 2026;16:04009.

**DOI:** 10.7189/jogh.16.1604009err1

**Published:** 2026-02-13

**Authors:** Journal of Global Health Editorial Team

Following the publication of the manuscript ‘Severe, primary, and incidental COVID-19 in hospitalised children, South Africa: 2020–2023’ on 30 January 2026, a style-related error occurred in Figure 1 and its associated title. The figure is a flowchart; therefore, the title has been corrected from ‘General definitions’ to ‘Flowchart’. Additionally, the general and specific definitions originally included within the figure were intended to be relocated to the Online Supplementary Document. However, this supporting text was inadvertently omitted from the supplement during the final stages of the production. 

The Online Supplementary Document has now been updated to include the complete set of definitions, and Figure 1 has been corrected accordingly. The editorial team regrets this error, takes full responsibility, and apologises for any inconvenience it may have caused to the readers or the authors.

These corrections have been made to the online and PDF version of the manuscript on 13 February 2026, following the authors’ notice on 3 February 2026.

