# Supplementary material for: Severe, primary, and incidental COVID-19 in hospitalised children, South Africa: 2020–2023
Source: J Glob Health. 2026 Jan 30;16:04009. doi: 10.7189/jogh.16.04009 (PMC12856961; doi:10.7189/jogh.16.04009)
Supplement: Online Supplementary Document [file jogh-16-04009-s001.pdf]

Supplement to: Goga A, Ramraj T, Cloete J, Mawela D, Waggie Z, Archary M, Chinniah K, Jeena P, Tabane NE, Van Zyl R, Reubenson G, Strehlau R, Feucht U, Reddy T, Mchunu N, Cawood S, Zühlke L, Webb K, Zar HJ, Donald KA, Scott C, Morrow BM, Aldersley T, du Plessis NM, Chetty T, Velaphi S, Dangor Z, Moore DP. Severe, primary, and incidental COVID-19 in hospitalised children, South Africa: 2020–2023. J Glob Health. 2026;16:04009.

General Definitions used in this paper

Acute Covid-19: Child with any new illness since 1 January 2020 and evidence of SARS-CoV-2 infection (RT-PCR, antigen test or serology positive) leading to hospitalisation (including all major and minor medical or surgical admissions), plus evidence of any SARS-CoV-2 exposure.

Child with SARS-CoV-2-related disease: Child with acute Covid-19 or MIS-C, or neonates with SARS-CoV-2 exposure.

Covid-19 variant periods: During data analysis a new variant period was designated as having started when 50% of genomes identified nationally comprised that variant. National data were used to identify variant periods because provincial-level variant data were incomplete. Individual-level genome testing to identify variants was not performed for this study. The periods are designated as: Ancestral, 03 March 2020-4 November 2020; Beta, 5 November 2020-2 June 2021; Delta, 3 June 2021-10 November 2021; Omicron, 11 November 2021-31 May 2023 (data cut-off for this analysis).

WHO MIS-C definition: Children and adolescents (age 0–19 years) with measured or self-reported fever ≥3 days and ***two or more of the following*** (i) Rash, or bilateral non-purulent conjunctivitis, or signs of mucocutaneous inflammation (involving mouth, hands or feet) (ii) Hypotension or shock (iii) Features of myocardial dysfunction - pericarditis, or valvulitis, or coronary abnormalities (clinical features, electrocardiogram (ECG), echocardiogram (ECHO) findings or laboratory markers such as elevated Troponin or N-terminal pro b-type natriuretic peptide (NT-proBNP)) (iv) Evidence of coagulopathy (clinical features or laboratory markers such as abnormal clotting factors, D-dimers, fibrinogen) (v) Acute gastrointestinal problems (such as diarrhoea, vomiting, or abdominal pain) **AND** Elevated markers of inflammation such as erythrocyte sedimentation rate (ESR), C-reactive protein (CRP) or procalcitonin (PCT) **AND** No other obvious microbial cause of inflammation, including bacterial sepsis, staphylococcal or streptococcal shock syndromes **AND** Evidence of SARS-CoV-2 infection: PCR, antigen or serology positive, or likely contact with SARS-CoV-2 positive persons.
SARS-CoV-2 exposure: Child with positive SARS-CoV-2 serology (IgM or IgG), or positive RT-PCR, or positive rapid antigen test (if site-specific rapid test approval in place), or likely contact with SARS-CoV-2 positive person, according to the hospital physician (including neonates born to SARS-CoV-2 positive mothers).

Underweight for age**:** Child with either a weight-for-age z-score (WAZ) < -2 if aged < 5 years, or body mass index (BMI) ≤ -2 z-score if aged 5-19 years at the time of presentation. The Fenton growth chart was used to improve assessment of growth (and underweight for age assessment) in children born preterm.

Specific definitions used to understand severity of Covid-19 in this paper, in order of appearance in the text

Severe disease: The child was hospitalised for primary or incidental Covid-19 or MIS-C with one or more of the following during the hospital admission: (i) Hypoxaemia, (ii) supplemental oxygen administration, (iii) hypotension (age-specific), (iv) vasopressor administration, (v) respiratory distress (age-specific), (vi) non-invasive or mechanical ventilation, or (viii) death.
Primary Covid-19: The child was hospitalised primarily because of symptoms relating to SARS-CoV-2 infection (including MIS-C)

Incidental Covid-19: The child was hospitalized for another primary reason, not Covid-19 or MIS-C. The child was tested for SARS-CoV-2 infection as a result of hospital testing protocols, and tested positive.
Severe Primary Covid-19: The child was hospitalised for primary Covid-19 (i.e., not an incidental SARS-CoV-2 infection) with one or more of the following during the hospital admission: (i) Hypoxaemia, (ii) supplemental oxygen administration, (iii) hypotension (age-specific), (iv) vasopressor administration, (v) respiratory distress (age-specific), (vi) non-invasive or mechanical ventilation, or (viii) death.

Supplementary Table S1: Study hospitals and distribution of enrolled children by study site

	**Ancestral**	**Beta**	**Delta**	**Omicron**	**Total**
	**N=350 (14.8%)**	**N=550 (23.3%)**	**N=603 (25.5%)**	**N=860 (36.4%)**	**N=2,363**
**Free State Province**					
Universitas - Academic Hospital	18 (5.1%)	21 (3.8%)	27 (4.5%)	0 (0.0%)	66 (2.8%)
Pelonomi Tertiary Hospital	2 (0.6%)	16 (2.9%)	9 (1.5%)	0 (0.0%)	27 (1.1%)
**Gauteng Province**					
Steve Biko Academic Pretoria	40 (11.4%)	75 (13.6%)	98 (16.3%)	246 (28.6%)	459 (9.4%)
Nelson Mandela Children's Hospital, Johannesburg	7 (2.0%)	3 (0.5%)	32 (5.3%)	10 (1.2%)	52 (2.2%)
Chris Hani Baragwanath Academic Hospital, Soweto	82 (23.4%)	116 (21.1%)	147 (24.4%)	327 (38.0%)	672 (28.4%)
Rahima Moosa Hospital	0 (0.0%)	0 (0.0%)	22 (3.6%)	4 (0.5%)	26 (1.1%)
George Mukhari Hospital	1 (0.3%)	55 (10.0%)	73 (12.1%)	127 (4.8%)	256 (10.8%)
**KZN Province**					
King Edward VIII Hospital	6 (1.7%)	36 (6.5%)	17 (2.8%)	12 (1.4%)	71 (3.0%)
Inkosi Albert Luthuli Hospital	21 (6.0%)	62 (11.3%)	27 (4.5%)	57 (6.6%)	167 (7.1%)
Mahatma Gandhi Memorial Hospital	7 (2.0%)	35 (6.4%)	23 (3.8%)	56 (6.5%)	121 (5.1%)
**Western Cape province**					
Red Cross War Memorial Children’s Hospital	136 (38.9%)	108 (19.7%)	126 (20.9%)	21 (2.4%)	391 (16.6%)
New Somerset Hospital	10 (2.9%)	5 (0.9%)	0 (0.0%)	0 (0.0%)	15 (0.6%)
Victoria Hospital	20 (5.7%)	17 (3.1%)	2 (0.3%)	0 (0.0%)	39 (1.7%)

Supplementary Text Box 1: Hospital characteristics and SARS-CoV-2 testing protocols during the study period

In KwaZulu-Natal (KZN), Free State and Western Cape Provinces: From March 2020 to May 2020, all children with fever or respiratory signs had SARS-CoV-2 PCR tests; from June 2020 to November 2022 all hospitalised children had PCR tests. Rapid antigen tests were used during this time, when available. From May 2023, children are not routinely tested for SARS-CoV-2 and clinician discretion was used for directed SARS-CoV-2 testing.

In Chris Hani Baragwanath Academic Hospital: All children from birth to 14 years of age with respiratory symptoms and/or critical illness from 31 March to 30 June 2020 were tested for SARS-CoV-2 infection; thereafter, all hospitalised children from 1 July 2020 to 3 July 2022 were tested. From 4 July 2022 onwards, testing was restricted to children requiring high care or critical care admission.

In Nelson Mandela Children's Hospital: From March 2020, all admissions of children from birth to 16 years were routinely tested with PCR testing (whether emergency or elective surgical admission, irrespective of symptomatology). From July 2022 no routine testing was required, and PCR testing was done at the discretion of attending clinicians.

In Rahima Moosa hospital: For children under 14 years of age, from March 2020, PCR testing was performed on children with acute respiratory symptoms requiring hospital admission; from July 2020, PCR testing was performed on all children requiring hospital admission; from July 2022, PCR testing was no longer routinely performed, but based on the discretion of treating clinicians. For neonates there was no routine testing, and only limited PCR testing at the discretion of treating clinicians. Access to antigen-testing was limited, and only conducted when rapid results were required.

In Steve Biko Academic Hospital all children were tested using SARS-CoV-2 PCR until December 2022. Thereafter only children with respiratory infections or diarrhoea were tested for SARS-CoV-2 infection.

In Kalafong Hospital: From 30 March to 21 July 2020, all children 1 month to 12 years with respiratory illnesses and children requiring intensive care support were tested using SARS-CoV-2 PCR; from 22 July 2020 to 31 December 2022, all children admitted to the hospital were routinely tested; from1 January 2023, clinician discretion was used to direct testing.

In Dr George Mukhari Academic Hospital: March 2020 to March 2023: All children 1 month to 12 years with respiratory symptoms, children requiring respiratory support and severely ill children were tested. All children requiring surgery (irrespective of symptoms) were tested. All neonates born to mothers with a positive test were tested. From April 2023 onwards: Clinician discretion was used to direct testing.

Supplementary Material: Results

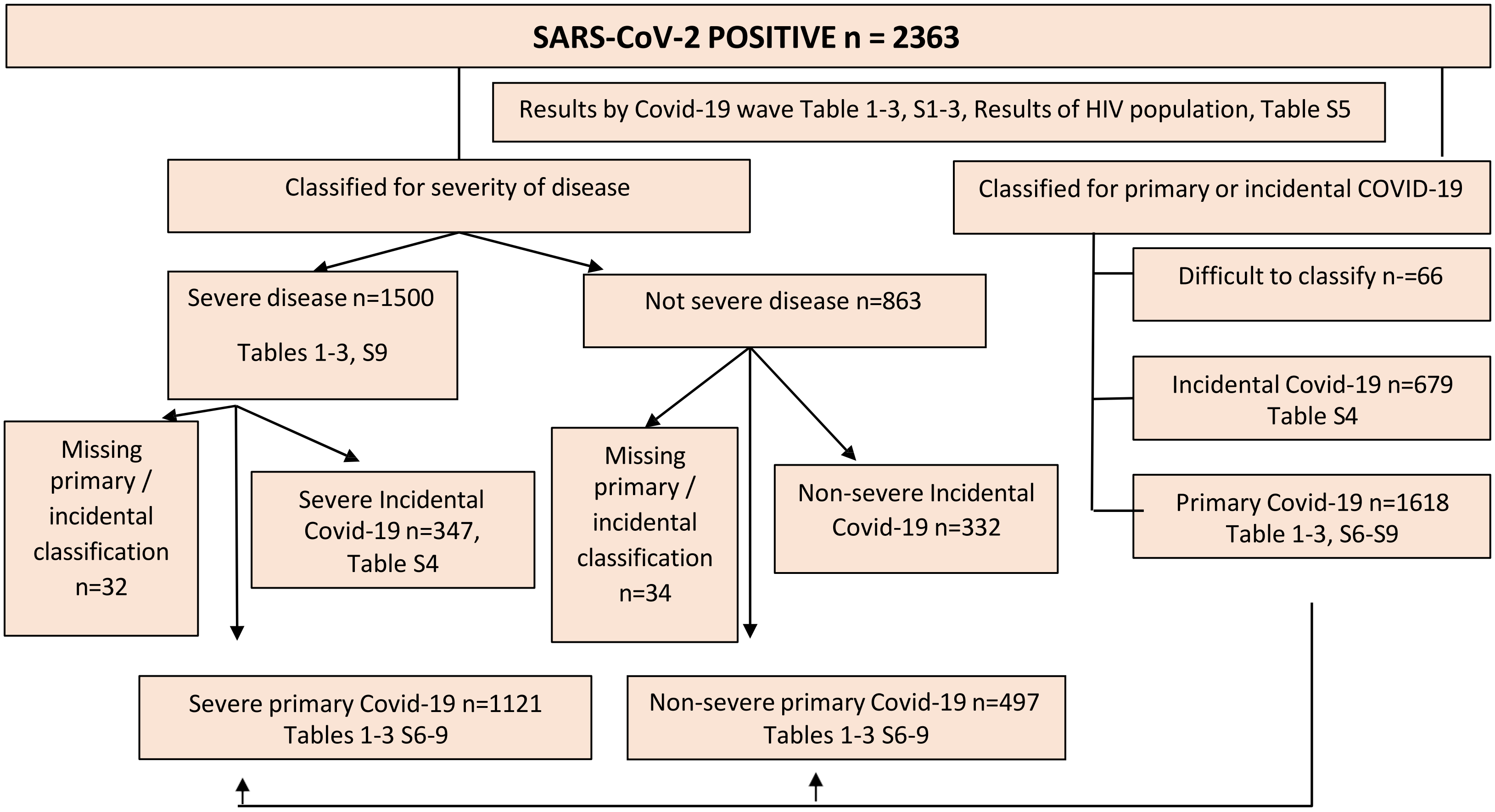

Supplementary Figure 1: Study population by severity of disease and Tables that describe their characteristics

Ninety-eight percent of participants tested SARS-CoV-2 PCR positive, 2.2% were SARS-CoV-2 rapid antigen test positive (some children had both tests (Table 1)). Of the 57 children eligible for vaccination against Covid-19 from October 2021 (>12 years old), 8 (14.0%) reported being vaccinated (Table 1). Of children with at least one comorbidity (n=731, 40%, Supplementary Table S2), 13.7% were reported to have a history of prematurity, 121 (5.4%) were infected with HIV, and 60 (2.6%) had TB, with no significant differences by variant period (Supplementary Table S2); 8% had a neurological disease (e.g. epilepsy); 5.0% had a pre-existing heart condition and this was significantly higher in the Ancestral versus the Omicron periods (p=0.002, Supplementary Table S2). Of those with available data, the proportion of children living in a household where Covid-19 had been diagnosed just before the child was hospitalised was highest in the Delta period (12.1%) and lowest in the Omicron period (4.5%), p<0.001, Table 1. HIV positivity did not differ significantly by variant period (Supplementary Table S2).

Supplementary Table S2: Symptoms, signs and comorbidities of all hospitalised children enrolled in the study with Covid-19, by wave

	**Ancestral 1 March 2020 until 4 November 2020**	**Beta 5 November 2020 -2nd June 2021**	**Delta 3rd June 2021-10 November 2021**	**Omicron 11 November 2021-31 May 2023**	**Total**	**p-value***	**Missing**
	**N=350 (14.8%)**	**N=550 (23.3%)**	**N=603 (25.5%)**	**N=860 (36.4%)**	N=2,363		
**Presenting symptoms on admission**							
**Respiratory**							
Cough	112 (38.9%)	139 (29.2%)	188 (33.5%)	245 (37.8%)	684 (34.7%)	0.50	171
Sore throat	14 (5.4%)	21 (5.4%)	30 (6.8%)	26 (3.3%)	91 (4.9%)	0.51	487
Fast or difficult breathing	157 (45.5%)	207 (39.0%)	201 (33.7%)	319 (37.3%)	884 (38.0%)	0.04	34
Rhinorrhoea	9 (5.8%)	24 (6.8%)	58 (13.1%)	119 (14.6%)	210 (11.9%)	0.001	596
Wheezing	51 (15.3%)	39 (7.6%)	51 (8.7%)	72 (8.5%)	213 (9.3%)	<0.001	79
Chest pain	13 (5.1%)	12 (3.0%)	11 (2.3%)	14 (1.9%)	50 (2.7%)	0.043	490
**Gastrointestinal**							
Abdominal pain	45 (14.8%)	53 (11.0%)	52 (9.9%)	61 (7.3%)	211 (9.8%)	0.002	216
Diarrhoea	64 (20.4%)	91 (18.2%)	93 (16.5%)	167 (19.9%)	415 (18.7%)	0.35	145
Vomiting	85 (26.9%)	122 (24.7%)	156 (27.4%)	219 (26.0%)	582 (26.2%)	0.78	143
Unable to drink	9 (5.2%)	22 (5.6%)	33 (7.1%)	48 (5.8%)	112 (6.0%)	0.571	498
**Mucocutaneous**							
Skin rash	37 (11.9%)	49 (9.8%)	55 (9.6%)	42 (5.0%)	183 (8.3%)	<0.001	146

	Ancestral 1 March 2020 until 4 November 2020	Beta 5 November 2020 -2nd June 2021	Delta 3rd June 2021- 10 November 2021	Omicron 11 November 2021-31 May 2023	Total	p-value*	Missing
Erythematous or sticky eyes or bilateral conjunctivitis	7 (5.0%)	9 (2.4%)	11 (2.5%)	25 (3.1%)	52 (2.9%)	0.42	593
Erythematous mouth or lips	2 (6.7%)	2 (2.2%)	4 (2.8%)	11 (2.9%)	19 (3.0%)	0.66	1720
Peeling or erythematous fingers, toes, palms, soles or oral / peripheral mucosal inflammation	8 (5.1%)	15 (4.0%)	25 (5.5%)	14 (1.7%)	62 (3.4%)	0.002	555
Skin ulcers	2 (1.4%)	3 (0.8%)	9 (2.0%)	3 (0.4%)	17 (1.0%)	0.033	601
**Musculoskeletal**							
Joint inflammation	5 (3.6%)	13 (3.6%)	17 (3.9%)	8 (1.0%)	43 (2.5%)	0.003	612
Arthralgia	9 (6.7%)	25 (6.9%)	16 (4.0%)	10 (1.2%)	60 (3.5%)	<0.001	652
Myalgia	16 (6.3%)	32 (7.7%)	31 (6.4%)	18 (2.2%)	97 (4.9%)	0.001	397
**Neurological**							
Neck stiffness	3 (1.7%)	9 (2.3%)	14 (3.1%)	7 (0.8%)	33 (1.8%)	0.030	514
Fatigue/malaise	42 (13.7%)	74 (15.4%)	88 (16.1%)	108 (13.0%)	312 (14.4%)	0.38	200
Seizures	5 (16.1%)	8 (9.0%)	13 (8.9%)	34 (8.9%)	60 (9.2%)	0.61	1714
Headache	19 (7.3%)	28 (7.2%)	42 (9.5%)	29 (3.7%)	118 (6.3%)	<0.01	480
Floppiness	8 (4.5%)	39 (9.8%)	34 (7.3%)	46 (5.6%)	127 (6.8%)	0.026	495
Paralysis	5 (2.8%)	12 (3.0%)	12 (2.6%)	9 (1.1%)	38 (2.0%)	0.072	489
Irritability	41 (13.0%)	76 (15.5%)	65 (11.5%)	120 (14.2%)	302 (13.6%)	0.26	150
Photophobia	3 (1.8%)	6 (1.7%)	9 (2.1%)	5 (0.6%)	23 (1.3%)	0.12	591
Anosmia	2 (1.0%)	2 (0.6%)	3 (0.7%)	4 (0.5%)	11 (0.6%)	0.89	652
**Other**							
Fever	155 (48.9%)	173 (34.6%)	219 (38.4%)	325 (38.4%)	872 (39.0%)	<0.001	129
Cervical lymphadenopathy	5 (3.1%)	13 (3.4%)	21 (4.6%)	16 (1.9%)	55 (3.0%)	0.61	540
Haemorrhage	6 (3.4%)	11 (2.8%)	11 (2.4%)	10 (1.2%)	38 (2.0%)	0.12	491
**Findings on clinical examination on admission**							
Respiratory rate Median (IQR)						<0.001-	

	Ancestral 1 March 2020 until 4 November 2020	Beta 5 November 2020 -2nd June 2021	Delta 3rd June 2021-10 November 2021	Omicron 11 November 2021-31 May 2023	Total	p-value*	Missing
0-28 days	48 (39.5-55)	43 (33.5-52)	42 (36-49)	42 (36-50)	42 (36-52)		45
29-365 days	40 (32-50)	42 (34-54)	40 (35-50)	40 (34-47)	40 (34-50)		76
1-5 years	34 (28-40)	31 (26-40)	30 (26-40)	32 (28-39)	32 (27-40)		105
>5-12 years	25 (22-31)	26 (22-32)	25 (22-30)	25 (22-30)	25 (22-31)		88
>12 years	24 (20-28)	20 (20-24)	22 (20-26)	20 (20-24)	20 (20-25)		40
Oxygen Saturation	97 (93-98)	96.5 (94-99)	97 (94-99)	96 (93-98)	96 (94-98)	<0.001	386
Hypotension	42 (18.3%)	45 (10.2%)	48 (9.6%)	24 (2.9%)	159 (7.9%)	<0.001	353
Tachycardia	150 (44.9%)	161 (31.6%)	229 (39.9%)	190 (22.4%)	730 (32.2%)	<0.001	96
Prolonged capillary refill time	8 (5.1%)	5 (1.4%)	24 (5.7%)	26 (3.2%)	63 (3.6%)	0.001	619
Pale/mottled skin	22 (9.2%)	43 (9.3%)	29 (5.8%)	22 (2.7%)	116 (5.7%)	<0.001	339
Cold peripheries	5 (3.0%)	8 (2.2%)	11 (2.6%)	21 (2.6%)	45 (2.6%)	0.95	603
Urinary output <2mL/kg/hr	2 (2.3%)	7 (3.1%)	10 (3.0%)	11 (1.5%)	30 (2.2%)	0.33	1004
**Presence of the following at any time during the hospitalisation**							
Ventilated	46 (21.1%)	60 (14.1%)	68 (13.7%)	48 (5.8%)	222 (11.3%)	<0.001	
Low oxygen saturation	82 (25.1%)	150 (28.7%)	161 (27.3%)	304 (35.4%)	697 (30.4%)	<0.001	
Hypotension	110 (50.0%)	181 (40.0%)	210 (42.0%)	282 (39.1%)	783 (41.3%)	0.033	
**Pre-existing comorbidities prior to hospitalisation**							
At least 1 comorbidity	68 (40.5%)	154 (41.3%)	199 (43.5%)	310 (37.3%)	731 (40.0%)	0.16	535
Prematurity	23 (14.6%)	49 (14.2%)	60 (13.8%)	104 (13.2%)	236 (13.7%)	0.96	641
Chronic neurological disease	35 (10.4%)	44 (8.5%)	50 (8.4%)	55 (6.4%)	184 (8.0%)	0.12	57
HIV positive	14 (4.2%)	24 (4.8%)	31 (5.4%)	52 (6.3%)	121 (5.4%)	0.47	139
Heart condition	25 (7.5%)	34 (6.6%)	30 (5.1%)	25 (2.9%)	114 (5.0%)	0.002	71
Diabetes mellitus	11 (5.7%)	14 (3.6%)	29 (6.2%)	28 (3.3%)	82 (4.3%)	0.69	472
Chronic lung condition	17 (5.2%)	14 (2.8%)	19 (3.3%)	19 (2.2%)	69 (3.1%)	0.075	120
Hypertension	8 (3.9%)	29 (7.2%)	18 (3.8%)	9 (1.1%)	64 (3.3%)	<0.001	443
Tuberculosis (TB)	10 (3.1%)	14 (2.8%)	22 (3.8%)	14 (1.6%)	60 (2.6%)	0.092	88

	Ancestral<br>1 March 2020 until<br>4 November 2020	Beta<br>5 November 2020 -2nd June 2021	Delta<br>3rd June 2021-10 November 2021	Omicron<br>11 November 2021-31 May 2023	Total	p-value*	Missing
Haematological disorder	6 (1.8%)	14 (2.8%)	17 (2.9%)	13 (1.5%)	50 (2.2%)	0.24	77
Asthma	7 (2.1%)	9 (1.8%)	10 (1.7%)	21 (2.5%)	47 (2.0%)	0.72	70
Chronic kidney disease	9 (2.7%)	16 (3.1%)	10 (1.7%)	8 (0.9%)	43 (1.9%)	0.019	71
Immune-related disease	4 (1.2%)	9 (1.8%)	16 (2.7%)	13 (1.5%)	42 (1.8%)	0.29	69
Cancer	4 (2.4%)	9 (2.4%)	8 (1.7%)	14 (1.7%)	35 (1.9%)	0.82	513
Inflammatory / rheumatological condition	3 (0.9%)	8 (1.6%)	8 (1.4%)	0 (0.0%)	19 (0.8%)	0.006	74
Respiratory disease other than tuberculosis	0 (0.0%)	1 (0.6%)	1 (0.4%)	12 (2.1%)	14 (1.3%)	0.012	1 311
Chronic liver disease	2 (0.6%)	4 (0.8%)	2 (0.3%)	6 (0.7%)	14 (0.6%)	0.78	74

Note: % calculated excluding missing data * test for trend across all periods

Supplementary Table S3: Clinical progress, most abnormal laboratory value* during the admission, management and hospital outcome of all hospitalised children enrolled in the study

	**Ancestral**<br>**March 2020 until 4 November 2020**	**Beta**<br>**5 November 2020 - 2nd June 2021**	**Delta**<br>**3rd June 2021-10 November 2021**	**Omicron**<br>**11 November 2021 -31 May 2023**	**Total**	**p-value***	**Missing**
	N=350<br>number<br>measurement	N=550<br>number<br>measurement	N=603<br>number<br>measurement	N=860<br>number<br>measurement	N=2,363<br>number<br>measurement		
Temperature	334<br>37 (36.5-37.7)	504<br>36.9 (36.5-37.55)	549<br>37 (36.5-37.6)	747<br>37 (36.7-37.7)	2134<br>37 (36.5-37.6)	0.013	229
Heart rate (beats/min)	338<br>140 (116-162)	506<br>133 (113-158)	557<br>140 (118-162)	755<br>145 (122-168)	2156<br>140 (118-163)	<0.001	207
Respiratory rate (breaths/min)	332<br>36 (26-48)	503<br>36 (25-50)	544<br>36 (26-47)	743<br>38 (29-48)	2122<br>37 (28-48)	0.023	241
Systolic blood pressure	220<br>104 (89-126)	453<br>110 (94-134)	500<br>106 (92-130.5)	721<br>101 (90-119)	1894<br>104 (91-125)	<0.001	469
Diastolic blood pressure	220<br>61.5 (51-78)	453<br>66 (53-88)	498<br>64 (54-85)	720<br>59.5 (52-71)	1891<br>62 (53-79)	<0.001	472
Percent oxygen saturation	321<br>97 (93-98)	507<br>97 (94-100)	568<br>97 (94-100)	760<br>96 (93-99)	2156<br>97 (94-99)	<0.001	207
Total WBC count (x10%)	233<br>11.27 (7.95-16.71)	307<br>9.59 (6.35-15.02)	387<br>10.96 (7.53-15.61)	516<br>11.325 (7.38-17.42)	1443<br>10.99 (7.27-16.37)	0.025	920
Neutrophils (count)	6.1 (2.4-15.5)	4.9 (2.6-10.3)	5.3 (2.6-10.3)	7.0 (3.2-16.7)	5.8 (2.8-12.2)	<0.001	
Total lymphocytes (count)	164<br>3.9 (1.9-6.6)	241<br>3.8 (2.0-7.4)	317<br>3.7 (2.1-6.6)	452<br>4.3 (2.2-11.0)	1174<br>4.0 (2.1-7.5)	0.002	1189
Platelets ($x10^9$/L)	233<br>323 (206-440)	309<br>343 (253-459)	406<br>354 (252-478)	509<br>393 (263-490)	1457<br>359 (251-474)	0.002	906
Creatinine (mg%)	206<br>38 (28-56)	271<br>38 (26-51)	356<br>38 (27-54)	513<br>37 (27-49)	1346<br>38 (27-51)	0.73	1017
Sodium (mmol/L)	217<br>136 (133-139)	280<br>137 (134-140)	366<br>137 (134-140)	526<br>138 (134-141)	1389<br>137 (134-140)	0.003	974
Potassium (mmol/L)	211<br>4.6 (4-5.3)	270<br>4.45 (3.9-5.1)	362<br>4.8 (4.1-5.4)	517<br>4.8 (4.1-5.5)	1360<br>4.7 (4-5.4)	<0.001	1003
Urea (BUN) (mg/dl)	136<br>4.6 (2.9-8.4)	190<br>3.95 (2.7-5.7)	244<br>4.55 (3-7.2)	495<br>495<br>4 (2.9-5.9)	1065<br>4.2 (2.9-6.4)	0.006	1298

	Ancestral<br>March 2020 until 4 November 2020	Beta<br>5 November 2020 - 2nd June 2021	Delta<br>3rd June 2021-10 November 2021	Omicron<br>11 November 2021 -31 May 2023	Total	p-value*	Missing
Glucose (mg/dl)	49<br>6.2 (4.7-6.9)	34<br>5.4 (4.8-6.7)	75<br>6.6 (4.8-17.9)	122<br>5.75 (4.4-7.5)	280<br>6 (4.7-7.8)	0.17	2083
PTT/APTR	41<br>27 (22.6-33)	66<br>26.55 (24.1-34.4)	98<br>25.35 (21-31.4)	84<br>24 (21.3-30.45)	289<br>26 (21.4-32.8)	0.063	2074
PT (seconds)	30<br>12.8 (11.9-14.5)	74<br>12.4 (11.4-13.9)	88<br>12.7 (11.7-14.35)	116<br>12.75 (11.4-14.975)	308<br>12.6 (11.5-14.45)	0.67	2055
INR	38<br>1.12 (1.03-1.23)	85<br>1.1 (1.04-1.21)	106<br>1.09 (1-1.18)	144<br>1.09 (1-1.245)	373<br>1.1 (1.02-1.23)	0.56	1990
Fibrinogen (mg/dl)	31<br>3.6 (1.16-5.90)	59<br>2.7 (2-4.2)	71<br>3.5 (2.2-4.8)	65<br>3.1 (2.2-4.4)	226<br>3.2 (2-4.5)	0.29	2137
Procalcitonin (ng/mL)	44<br>1.52 (0.07-13.95)	56<br>0.845 (0.055-5.59)	82<br>2.235 (0.16-22.69)	92<br>0.49 (0.16-5.015)	274<br>1.025 (0.14-8.65)	0.28	2089
CRP (mg/L)	188<br>25 (4-105.5)	242<br>17 (3-76)	326<br>14 (3-67)	446<br>16 (4-56)	1202<br>16 (3-69)	0.17	1161
D-dimer (ng/L)	49<br>3 (1-10.67)	74<br>2.525 (.71-6.87)	88<br>1.105 (.43-3.05)	126<br>1.32 (.49-6.29)	337<br>1.69 (.56-6)	0.002	2026
IL-6 (pg/mL)	1<br>48.7 (48.7-48.7)	3<br>4.6 (3.91-5.8)	0	0	4<br>5.2 (4.255-27.25)	0.18	2359
Pro-BNP (pg/mL)	16<br>670.5 (40.5-17535.5)	17<br>144 (36-921)	15<br>(5.8-550)	8<br>219.5 (44-2392)	56<br>207 (29-1701.5)	0.56	2307
Troponin (ng/mL)	32<br>11.5 (10-59)	43<br>14 (10-45)	59<br>(10-56)	81<br>10 (10-12)	215<br>10 (10-37)	0.015	2148
Creatine kinase (U/L)	20<br>108.5 (55-228)	40<br>129 (49.5-401)	69<br>(52-286)	98<br>68.5 (34-167)	227<br>91 (41-221)	0.082	2136
**Bacterial pathogen testing**						<0.001	2
Positive	39 (11.1%)	62 (11.3%)	65 (10.8%)	64 (7.5%)	230 (9.7%)		
Negative	151 (43.1%)	149 (27.1%)	229 (38.0%)	300 (34.9%)	829 (35.1%)		
Not Done	160 (45.7%)	339 (61.6%)	308 (51.2%)	495 (57.6%)	1,302 (55.1%)		
**Chest X-ray performed**						<0.001	4
Yes	164 (47.0%)	209 (38.0%)	195 (32.3%)	195 (22.8%)	763 (32.3%)		
No	167 (47.9%)	286 (52.0%)	357 (59.2%)	615 (71.8%)	1,425 (60.4%)		
Unknown	18 (5.2%)	55 (10.0%)	51 (8.5%)	47 (5.5%)	171 (7.2%)		
**CT performed**						0.001	26
Yes	12 (3.5%)	20 (3.7%)	23 (3.9%)	30 (3.5%)	85 (3.6%)		

	Ancestral March 2020 until 4 November 2020	Beta 5 November 2020 - 2nd June 2021	Delta 3rd June 2021-10 November 2021	Omicron 11 November 2021 -31 May 2023	Total	p-value*	Missing
No	324 (94.7%)	488 (89.5%)	543 (91.4%)	805 (94.0%)	2,160 (92.4%)		
Unknown	6 (1.8%)	37 (6.8%)	28 (4.7%)	21 (2.5%)	92 (3.9%)		
**Echocardiography (ECHO) performed**						<0.001	24
Yes	40 (11.5%)	39 (7.2%)	40 (6.8%)	26 (3.0%)	145 (6.2%)		
No	296 (85.1%)	459 (84.4%)	520 (88.1%)	754 (88.0%)	2,029 (86.7%)		
Unknown	12 (3.4%)	46 (8.5%)	30 (5.1%)	77 (9.0%)	165 (7.1%)		
**On that echocardiogram were there:**							
**Features of myocardial dysfunction**						0.11	28
Yes	8 (24.2%)	12 (34.3%)	4 (12.5%)	3 (12.5%)	27 (21.8%)		
No	20 (60.6%)	13 (37.1%)	19 (59.4%)	17 (70.8%)	69 (55.6%)		
Unknown	5 (15.2%)	10 (28.6%)	9 (28.1%)	4 (16.7%)	28 (22.6%)		
**Features of pericarditis**						0.94	124
Yes	1 (17%)	2 (20%)	1 (33%)	1 (33%)	5 (23%)		
No	5 (83%)	7 (70%)	2 (67%)	2 (67%)	16 (73%)		
Unknown	0 (0%)	1 (10%)	0 (0%)	0 (0%)	1 (5%)		
**Features of valvulitis**						0.62	124
Yes	0 (0%)	1 (11%)	0 (0%)	1 (33%)	2 (10%)		
No	6 (100%)	7 (78%)	3 (100%)	2 (67%)	18 (86%)		
Unknown	0 (0%)	1 (11%)	0 (0%)	0 (0%)	1 (5%)		
**Coronary abnormalities**						0.27	123
Yes	5 (83%)	4 (40%)	0 (0%)	2 (67%)	11 (50%)		
No	1 (17%)	5 (50%)	3 (100%)	1 (33%)	10 (45%)		
Unknown	0 (0%)	1 (10%)	0 (0%)	0 (0%)	1 (5%)		
**Intravenous fluids given**						<0.001	79
Yes	169 (53.0%)	255 (48.5%)	264 (45.2%)	369 (43.2%)	1,057 (46.3%)		
No	142 (44.5%)	223 (42.4%)	261 (44.7%)	467 (54.6%)	1,093 (47.9%)		
Unknown	8 (2.5%)	48 (9.1%)	59 (10.1%)	19 (2.2%)	134 (5.9%)		
**Corticosteroid given**						<0.001	83
Yes	43 (13.5%)	77 (14.6%)	89 (15.3%)	83 (9.7%)	292 (12.8%)		
No	271 (85.0%)	433 (82.3%)	472 (81.4%)	767 (89.7%)	1,943 (85.2%)		
Unknown	5 (1.6%)	16 (3.0%)	19 (3.3%)	5 (0.6%)	45 (2.0%)		

	Ancestral March 2020 until 4 November 2020	Beta 5 November 2020 - 2nd June 2021	Delta 3rd June 2021-10 November 2021	Omicron 11 November 2021 -31 May 2023	Total	p-value*	Missing
**IV immune globulin given**						<0.001	80
Yes	21 (6.6%)	22 (4.2%)	34 (5.8%)	9 (1.1%)	86 (3.8%)		
No	293 (91.8%)	493 (93.7%)	532 (91.4%)	843 (98.5%)	2,161 (94.7%)		
Unknown	5 (1.6%)	11 (2.1%)	16 (2.7%)	4 (0.5%)	36 (1.6%)		
**Antibiotic given**						0.075	80
Yes	239 (75.2%)	371 (70.4%)	409 (70.2%)	613 (71.7%)	1,632 (71.5%)		
No	76 (23.9%)	146 (27.7%)	157 (26.9%)	234 (27.4%)	613 (26.9%)		
Unknown	3 (0.9%)	10 (1.9%)	17 (2.9%)	8 (0.9%)	38 (1.7%)		
**Antifungal agent given**						<0.001	396
Yes	26 (11.8%)	50 (11.7%)	40 (8.4%)	46 (5.5%)	162 (8.3%)		
No	192 (87.3%)	368 (86.4%)	423 (88.3%)	784 (93.7%)	1,767 (90.1%)		
Unknown	2 (0.9%)	8 (1.9%)	16 (3.3%)	7 (0.8%)	33 (1.7%)		
**Systemic anticoagulation given**						<0.001	84
Yes	30 (9.4%)	45 (8.6%)	52 (8.9%)	40 (4.7%)	167 (7.3%)		
No	286 (89.9%)	471 (90.1%)	511 (87.8%)	809 (94.5%)	2,077 (91.1%)		
Unknown	2 (0.6%)	7 (1.3%)	19 (3.3%)	7 (0.8%)	35 (1.5%)		
**Oxygen supplementation therapy given**						0.16	449
Yes	73 (39.7%)	141 (33.7%)	151 (31.9%)	299 (35.7%)	664 (34.7%)		
No	107 (58.2%)	269 (64.2%)	311 (65.6%)	530 (63.3%)	1,217 (63.6%)		
Unknown	4 (2.2%)	9 (2.1%)	12 (2.5%)	8 (1.0%)	33 (1.7%)		
**Number of days of oxygen therapy**	5 (2-8)	5 (2-8)	4 (2-8)	3 (2-5)	4 (2-7)	0.009	124
**Inotropes/vasopressors given**						0.006	
Yes	13 (5.9%)	10 (2.3%)	22 (4.3%)	18 (2.2%)	63 (3.1%)		361
No	202 (91.8%)	414 (94.7%)	472 (92.9%)	809 (96.7%)	1,897 (94.8%)		
Unknown	5 (2.3%)	13 (3.0%)	14 (2.8%)	10 (1.2%)	42 (2.1%)		
**Child admitted to ICU**						<0.001	
Yes	20 (33.9%)	26 (15.8%)	57 (21.1%)	38 (6.9%)	141 (13.5%)		1313
No	39 (66.1%)	139 (84.2%)	213 (78.9%)	513 (93.1%)	904 (86.5%)		
**Child admitted to ICU or high dependency unit admission**						<0.001	12

	Ancestral March 2020 until 4 November 2020	Beta 5 November 2020 - 2nd June 2021	Delta 3rd June 2021-10 November 2021	Omicron 11 November 2021 -31 May 2023	Total	p-value*	Missing
Yes	78 (22.3%)	97 (17.7%)	131 (21.9%)	115 (13.4%)	421 (17.9%)		
No	269 (77.1%)	440 (80.4%)	457 (76.5%)	736 (85.8%)	1,902 (80.9%)		
Unknown	2 (0.6%)	10 (1.8%)	9 (1.5%)	7 (0.8%)	28 (1.2%)		
**Number of days in ICU**	5.5 (4-10)	6 (4-10)	4 (3-7)	4 (3-6)	5 (3-8)	0.003	135
**Non-invasive ventilation (e.g. BiPAP/CPAP)**						<0.001	
Yes	21 (9.5%)	32 (7.3%)	34 (6.7%)	15 (1.8%)	102 (5.1%)		360
No	196 (88.7%)	392 (89.7%)	457 (90.1%)	815 (97.3%)	1,860 (92.9%)		
Unknown	4 (1.8%)	13 (3.0%)	16 (3.2%)	8 (1.0%)	41 (2.0%)		
**Invasive ventilation**						<0.001	
Yes	28 (12.5%)	38 (8.7%)	42 (8.3%)	37 (4.4%)	145 (7.2%)		358
No	193 (86.2%)	387 (88.6%)	449 (88.7%)	794 (94.7%)	1,823 (90.9%)		
Unknown	3 (1.3%)	12 (2.7%)	15 (3.0%)	7 (0.8%)	37 (1.8%)		
**Duration of ventilation in days**	4.5 (2-11)	7 (5-10)	4 (3-5)	5 (2-9)	5 (3-8)	0.016	30
**Blood transfusion given**						<0.001	
Yes	25 (11.3%)	55 (12.6%)	58 (11.4%)	62 (7.4%)	200 (10.0%)		359
No	191 (86.4%)	370 (84.9%)	434 (85.3%)	769 (91.8%)	1,764 (88.0%)		
Unknown	5 (2.3%)	11 (2.5%)	17 (3.3%)	7 (0.8%)	40 (2.0%)		
**Outcome**						<0.001	3
Discharged alive	300 (86.0%)	473 (86.2%)	537 (89.2%)	796 (92.6%)	2,106 (89.2%)		
Hospitalised	0 (0.0%)	1 (0.2%)	4 (0.7%)	6 (0.7%)	11 (0.5%)		
Transferred to other facility	26 (7.4%)	53 (9.7%)	42 (7.0%)	33 (3.8%)	154 (6.5%)		
Death	20 (5.7%)	17 (3.1%)	16 (2.7%)	23 (2.7%)	76 (3.2%)		
Left against medical advice	1 (0.3%)	0 (0.0%)	0 (0.0%)	0 (0.0%)	1 (0.0%)		
Unknown	2 (0.6%)	5 (0.9%)	3 (0.5%)	2 (0.2%)	12 (0.5%)		

*test for trend across all periods

Supplementary Table S4: Socio-demographic characteristics of enrolled in the study by primary or incidental Covid-19

	Primary Covid-19*						
	Ancestral	Beta	Delta	Omicron	Total	p-value	Missing
	N=275 (17%)	N=357 (22.1%)	N=399 (24.7%)	N=587 (36.3%)	N=1,618		0
**Study selection**						<0.001	381
Retrospective	2 (1.6%)	3 (1.2%)	93 (32.0%)	428 (74.8%)	526 (42.5%)		
Prospective	126 (98.4%)	243 (98.8%)	198 (68.0%)	144 (25.2%)	711 (57.5%)		
**Province**						<0.001	0
Free State	13 (4.7%)	11 (3.1%)	13 (3.3%)	0 (0.0%)	37 (2.3%)		
Gauteng	88 (32.0%)	149 (41.7%)	239 (59.9%)	509 (86.7%)	985 (60.9%)		
KwaZulu-Natal	27 (9.8%)	86 (24.1%)	39 (9.8%)	63 (10.7%)	215 (13.3%)		
Western Cape	147 (53.5%)	111 (31.1%)	108 (27.1%)	15 (2.6%)	381 (23.5%)		
**Severe**	193 (70.2%)	257 (72.0%)	264 (66.2%)	407 (69.3%)	1,121 (69.3%)	0.37	0
**Child's HIV status**						0.065	61
Positive (on ART)	7 (3.1%)	7 (2.5%)	13 (3.9%)	25 (4.7%)	52 (3.8%)		
Negative	255 (95.9%)	324 (96.4%)	364 (95.5%)	533 (92.9%)	1,476 (94.8%)		
Positive (not on ART)	4 (1.8%)	4 (1.4%)	4 (1.2%)	16 (3.0%)	28 (2.0%)		
**Age category**						<0.001	25
0-28 days	30 (11.0%)	32 (9.1%)	32 (8.2%)	35 (6.0%)	129 (8.1%)		
29-365 days	63 (23.2%)	123 (35.1%)	125 (32.0%)	237 (40.9%)	548 (34.4%)		
1-5 years	79 (29.0%)	83 (23.7%)	97 (24.8%)	180 (31.0%)	439 (27.6%)		
5-12 years	64 (23.5%)	57 (16.3%)	85 (21.7%)	111 (19.1%)	317 (19.9%)		
>12 years	36 (13.2%)	55 (15.7%)	52 (13.3%)	17 (2.9%)	160 (10.0%)		
**History of Covid-19 in the household**						<0.001	253
**Yes**	30 (12.7%)	29 (11.1%)	44 (14.1%)	27 (4.9%)	130 (9.5%)		
**No**	206 (87.3%)	233 (88.9%)	268 (85.9%)	528 (95.1%)	1,235 (90.5%)		
**Has the child received a Covid-19 vaccine?**						0.69	647

	Incidental Covid-19*						
	Ancestral	Beta	Delta	Omicron	Total	p-value†	Missing
	N=64 (9.4%)	N=183 (27%)	N=195 (28.7%)	N=237 (34.9%)	N=679		
**Study selection**						<0.001	42
Retrospective	55 (98.2%)	169 (98.8%)	120 (67.0%)	79 (34.2%)	423 (66.4%)		
Prospective	1 (1.8%)	2 (1.2%)	59 (33.0%)	152 (65.8%)	214 (33.6%)		
**Province**						<0.001	
Free State	7 (10.9%)	26 (14.2%)	23 (11.8%)	0 (0.0%)	56 (8.2%)		0
Gauteng	42 (65.6%)	98 (53.6%)	132 (67.7%)	178 (75.1%)	450 (66.3%)		
KwaZulu-Natal	7 (10.9%)	47 (25.7%)	24 (12.3%)	53 (22.4%)	131 (19.3%)		
Western Cape	8 (12.5%)	12 (6.6%)	16 (8.2%)	6 (2.5%)	42 (6.2%)		
**Severe**	33 (51.6%)	98 (53.6%)	94 (48.2%)	122 (51.5%)	347 (51.1%)	0.77	0
**Child's HIV status**						0.29	74
Positive (on ART)	3 (5.3%)	12 (8.1%)	13 (7.2%)	8 (3.7%)	36 (6.0%)		
Negative	54 (94.7%)	137 (91.9%)	168 (92.8%)	210 (96.3%)	569 (94.0%)		
Positive (not on ART)							
**Age category**						0.002	10
0-28 days	12 (18.8%)	16 (9.0%)	20 (10.5%)	21 (8.9%)	69 (10.3%)		
29-365 days	8 (12.5%)	26 (14.6%)	36 (18.9%)	43 (18.1%)	113 (16.9%)		
1-5 years	17 (26.6%)	31 (17.4%)	37 (19.5%)	73 (30.8%)	158 (23.6%)		
5-12 years	14 (21.9%)	54 (30.3%)	64 (33.7%)	65 (27.4%)	197 (29.4%)		
>12 years	13 (20.3%)	51 (28.7%)	33 (17.4%)	35 (14.8%)	132 (19.7%)		
**History of Covid-19 in the household**						0.32	180
**Yes**	3 (6.4%)	9 (8.4%)	10 (7.5%)	8 (3.8%)	30 (6.0%)		
**No**	44 (93.6%)	98 (91.6%)	123 (92.5%)	204 (96.2%)	469 (94.0%)		
**Has the child received a Covid-19 vaccine?**						0.24	201

	Primary Covid-19*							Incidental Covid-19*						
	Ancestral	Beta	Delta	Omicron	Total	p-value	Missing	Ancestral	Beta	Delta	Omicron	Total	p-value†	Missing
**Yes**	0 (0.0%)	0 (0.0%)	1 (0.5%)	3 (0.6%)	4 (0.4%)			0 (0.0%)	0 (0.0%)	0 (0.0%)	3 (1.5%)	3 (0.6%)		
**No**	80 (100.0%)	172 (100.0%)	211 (99.5%)	504 (99.4%)	967 (99.6%)			32 (100.0%)	107 (100.0%)	138 (100.0%)	198 (98.5%)	475 (99.4%)		
**Has the mother received a Covid-19 vaccine?**						<0.001	845						0.002	322
**Yes**	16 (30.2%)	10 (9.0%)	29 (17.5%)	164 (37.0%)	219 (28.3%)			1 (5.6%)	13 (19.4%)	23 (22.5%)	62 (36.5%)	99 (27.7%)		
**No**	80 (100.0%)	172 (100.0%)	211 (99.5%)	504 (99.4%)	967 (99.6%)			17 (94.4%)	54 (80.6%)	79 (77.5%)	108 (63.5%)	258 (72.3%)		
**Has the father received a Covid-19 vaccine?**						<0.001	960						0.079	386
Yes	14 (32.6%)	14 (13.5%)	25 (17.1%)	125 (34.2%)	178 (27.1%)			1 (7.1%)	10 (16.7%)	12 (13.8%)	34 (25.8%)	57 (19.5%)		
No	29 (67.4%)	90 (86.5%)	121 (82.9%)	240 (65.8%)	480 (72.9%)			13 (92.9%)	50 (83.3%)	75 (86.2%)	98 (74.2%)	236 (80.5%)		
**Nutritional status**						0.009	182						0.28	142
Normal/Overweight	186 (77.8%)	198 (68.0%)	267 (74.6%)	372 (67.9%)	1,023 (71.2%)			46 (82.1%)	96 (79.3%)	109 (71.7%)	163 (78.4%)	414 (77.1%)		
Underweight	53 (22.2%)	93 (32.0%)	91 (25.4%)	176 (32.1%)	413 (28.8%)			10 (17.9%)	25 (20.7%)	43 (28.3%)	45 (21.6%)	123 (22.9%)		
**Has the child been admitted to hospital in the last 3 months**						0.47	567						0.78	160
Yes	12 (11.9%)	28 (15.3%)	39 (15.6%)	92 (17.8%)	171 (16.3%)			7 (17.1%)	18 (14.4%)	28 (18.8%)	32 (15.7%)	85 (16.4%)		
No	89 (88.1%)	155 (84.7%)	211 (84.4%)	425 (82.2%)	880 (83.7%)			34 (82.9%)	107 (85.6%)	121 (81.2%)	172 (84.3%)	434 (83.6%)		
**History of Covid-19 in the previous 4 weeks prior to current illness**						0.013	477						0.001	115
Yes – Lab confirmed	6 (5.3%)	11 (5.4%)	12 (4.5%)	8 (1.4%)	37 (3.2%)			4 (7.7%)	16 (11.8%)	6 (3.8%)	3 (1.4%)	29 (5.1%)		
Yes – Clinically diagnosed	0 (0.0%)	1 (0.5%)	2 (0.7%)	0 (0.0%)	3 (0.3%)			0 (0.0%)	2 (1.5%)	2 (1.3%)	1 (0.5%)	5 (0.9%)		
No	108 (94.7%)	193 (94.1%)	253 (94.8%)	547 (98.6%)	1,101 (96.5%)			48 (92.3%)	118 (86.8%)	148 (94.9%)	216 (98.2%)	530 (94.0%)		
**History of any respiratory infection in the previous 4 weeks prior to current hospitalisation**						0.098	486						0.69	120
Yes	5 (4.5%)	10 (5.0%)	17 (6.5%)	16 (2.9%)	48 (4.2%)			0 (0.0%)	1 (0.8%)	3 (1.9%)	3 (1.4%)	7 (1.3%)		
No	105 (95.5%)	192 (95.0%)	243 (93.5%)	544 (97.1%)	1,084 (95.8%)			48 (100.0%)	132 (99.2%)	153 (98.1%)	219 (98.6%)	552 (98.7%)		
**SARS-CoV-2 RT-PCR**						<0.001	0						0.21	0
Positive	273 (99.3%)	354 (99.2%)	394 (98.7%)	562 (95.7%)	1,583 (97.8%)			64 (100.0%)	183 (100.0%)	194 (99.5%)	231 (97.5%)	672 (99.0%)		
Negative	2 (0.7%)	1 (0.3%)	2 (0.5%)	0 (0.0%)	5 (0.3%)			0 (0.0%)	0 (0.0%)	0 (0.0%)	1 (0.4%)	1 (0.1%)		
Not Done	0 (0.0%)	2 (0.6%)	3 (0.8%)	25 (4.3%)	30 (1.9%)			0 (0.0%)	0 (0.0%)	1 (0.5%)	5 (2.1%)	6 (0.9%)		
**SARS-CoV-2 rapid antigen test**						<0.001	0						0.21	0

	Primary Covid-19*								Incidental Covid-19*						
	**Ancestral**	**Beta**	**Delta**	**Omicron**	**Total**	**p-value**	**Missing**		**Ancestral**	**Beta**	**Delta**	**Omicron**	**Total**	**p-value†**	**Missing**
Positive	0 (0.0%)	10 (2.8%)	4 (1.0%)	26 (4.4%)	40 (2.5%)				0 (0.0%)	2 (1.1%)	2 (1.0%)	8 (3.4%)	12 (1.8%)		
Negative	1 (0.4%)	1 (0.3%)	4 (1.0%)	1 (0.2%)	7 (0.4%)				0 (0.0%)	1 (0.5%)	3 (1.5%)	1 (0.4%)	5 (0.7%)		
**Not Done**	274 (99.6%)	346 (96.9%)	391 (98.0%)	560 (95.4%)	1,571 (97.1%)				64 (100.0%)	180 (98.4%)	190 (97.4%)	228 (96.2%)	662 (97.5%)		
**SARS-CoV-2 ELISA test**						0.005	1	**SARS-CoV-2 ELISA test**						0.52	0
Positive	3 (1.1%)	2 (0.6%)	4 (1.0%)	5 (0.9%)	14 (0.9%)				0 (0.0%)	1 (0.5%)	1 (0.5%)	3 (1.3%)	5 (0.7%)		
Negative	4 (1.5%)	8 (2.2%)	10 (2.5%)	36 (6.1%)	58 (3.6%)				1 (1.6%)	3 (1.6%)	4 (2.1%)	10 (4.2%)	18 (2.7%)		
Not Done	268 (97.5%)	347 (97.2%)	385 (96.5%)	545 (93.0%)	1,545 (95.5%)				63 (98.4%)	179 (97.8%)	190 (97.4%)	224 (94.5%)	656 6.6 %)		

*66 children were difficult to adjudicate as having primary or incidental Covid-19 and are thus excluded from this table †test for trend across all periods

Supplementary Table S5. Association between Covid-19 and HIV in hospitalised children

	**CLHIV Number/total (%)**	**Children without HIV Number/total (%)**	**p-values**
Received oxygen*	41/109 (37.6)	592/1644 (36.0)	0.76
Invasive or non-invasive ventilation *	14/110 (12.7)	195/1733/ (11.3)	0.641
Severe disease*	88/121 (72.7)	1320/2104 (62.7)	0.026

	**CLHIV Number/total (%)**	**Children living with HIV not on ART Number/total (%)**	
Supplemental oxygen administration*	22/68 (32.4)	19/41 (46.3)	0.158
Invasive or non-invasive ventilation*	6/70 (8.6)	8/40 (20.0)	0.134
Severe disease*	51/80 (63.8)	37/41 (90.2)	0.002

*The total is not the same for each row within each group because of missing data

Supplementary Table S6: Symptoms, signs and comorbidities recorded in the medical record at any time during the hospitalisation amongst children with primary Covid-19, by wave

	Ancestral	Beta	Delta	Omicron	Total	p-value*	Missing†
	N=275	N=357	N=399	N=587	N=1,618		0
	number (%)	number (%)	number (%)	number (%)	number (%)		
**Respiratory symptoms**							
Cough	108 (45.8%)	134 (40.7%)	173 (45.6%)	327 (56.5%)	742 (48.7%)	<0.001	95
Sore throat	12 (5.8%)	19 (7.6%)	23 (8.3%)	24 (4.5%)	78 (6.1%)	0.12	348
Fast breathing	109 (42.7%)	135 (40.1%)	140 (36.6%)	195 (33.5%)	579 (37.2%)	0.046	62
Difficulty breathing	101 (37.8%)	136 (39.0%)	128 (32.2%)	252 (43.1%)	617 (38.6%)	0.008	20
Rhinorrhoea	9 (8.4%)	22 (10.6%)	56 (20.4%)	115 (20.4%)	202 (17.5%)	<0.001	464
Wheezing	47 (18.0%)	38 (11.2%)	50 (12.8%)	68 (11.6%)	203 (12.9%)	0.051	42
Chest pain	12 (6.0%)	10 (3.8%)	7 (2.3%)	13 (2.5%)	42 (3.3%)	0.082	337
**Gastro-intestinal symptoms**							
Abdominal pain	35 (14.5%)	30 (9.5%)	38 (11.0%)	42 (7.3%)	145 (9.8%)	0.013	142
Diarrhoea	56 (22.5%)	80 (24.2%)	84 (22.2%)	153 (26.4%)	373 (24.3%)	0.43	81
Vomiting	72 (28.7%)	103 (31.4%)	128 (33.4%)	180 (31.0%)	483 (31.3%)	0.66	76
Unable to drink	8 (6.7%)	17 (7.3%)	20 (7.0%)	36 (6.3%)	81 (6.7%)	0.96	414
**Mucocutaneous symptoms**							
Rash	31 (12.5%)	38 (11.5%)	46 (12.0%)	34 (5.9%)	149 (9.7%)	0.001	79
Erythematous or sticky eyes or bilateral conjunctivitis	7 (7.1%)	9 (4.0%)	9 (3.3%)	20 (3.5%)	45 (3.9%)	0.36	452
Erythematous mouth or lips	2 (8.0%)	2 (2.9%)	4 (4.0%)	10 (3.1%)	18 (3.5%)	0.62	1,102
Peeling or erythematous fingers, toes, palms, soles or oral /peripheral mucosal inflammation	5 (4.5%)	10 (4.4%)	15 (5.4%)	9 (1.6%)	39 (3.3%)	0.015	432
**Musculoskeletal symptoms**							
**Inflamed joints**	3 (3.1%)	4 (1.8%)	6 (2.3%)	3 (0.5%)	16 (1.4%)	0.078	473
Arthralgia	7 (7.4%)	9 (4.1%)	4 (1.7%)	6 (1.1%)	26 (2.3%)	<0.001	501
Myalgia	12 (6.0%)	17 (6.2%)	22 (6.9%)	11 (1.9%)	62 (4.6%)	0.002	257
Skin ulcers	2 (2.0%)	3 (1.3%)	6 (2.2%)	1 (0.2%)	12 (1.0%)	0.031	459
Neurological symptoms							

	Ancestral	Beta	Delta	Omicron	Total	p-value*	Missing†
	**N=275**	**N=357**	**N=399**	**N=587**	**N=1,618**		**0**
	**number (%)**	**number (%)**	**number (%)**	**number (%)**	**number (%)**		
Stiff neck	3 (2.5%)	3 (1.3%)	8 (2.8%)	4 (0.7%)	18 (1.5%)	0.082	407
Fatigue / malaise	34 (14.1%)	48 (15.1%)	64 (17.5%)	89 (15.5%)	235 (15.7%)	0.70	120
Seizures	5 (19.2%)	6 (8.8%)	10 (9.9%)	28 (8.7%)	49 (9.5%)	0.36	1,100
Headache	16 (7.8%)	14 (5.5%)	31 (11.1%)	19 (3.5%)	80 (6.2%)	<0.001	334
Floppiness	6 (4.8%)	22 (9.1%)	22 (7.6%)	36 (6.3%)	86 (7.0%)	0.38	393
Paralysis	2 (1.7%)	3 (1.2%)	5 (1.7%)	3 (0.5%)	13 (1.1%)	0.35	397
Irritability	37 (15.0%)	57 (17.8%)	52 (13.7%)	96 (16.6%)	242 (15.9%)	0.46	92
Photophobia	2 (1.8%)	5 (2.4%)	4 (1.5%)	3 (0.5%)	14 (1.2%)	0.17	468
Hyposmia / anosmia	2 (1.3%)	2 (0.9%)	2 (0.8%)	2 (0.4%)	8 (0.7%)	0.63	465
Ageusia	0 (0.0%)	2 (3.1%)	1 (1.1%)	3 (1.0%)	6 (1.2%)	0.49	1,126
**Other symptoms**							
Fever	139 (56.0%)	153 (46.4%)	190 (50.3%)	278 (48.0%)	760 (49.5%)	0.10	83
Cervical lymphadenopathy	4 (3.6%)	7 (3.0%)	12 (4.2%)	9 (1.6%)	32 (2.7%)	0.13	421
Haemorrhage	4 (3.3%)	2 (0.9%)	5 (1.7%)	3 (0.5%)	14 (1.2%)	0.051	406
**Signs:**							
Hypotension	39 (22.9%)	33 (12.0%)	36 (11.3%)	18 (3.2%)	126 (9.4%)	<0.001	283
Tachycardia (age-appropriate)	131 (49.8%)	126 (38.1%)	186 (48.2%)	158 (27.2%)	601 (38.5%)	<0.001	57
Prolonged capillary refill time	7 (6.4%)	4 (1.9%)	15 (5.7%)	23 (4.1%)	49 (4.3%)	0.15	480
Pale/mottled skin	17 (9.5%)	28 (9.6%)	21 (6.4%)	12 (2.1%)	78 (5.7%)	<0.001	252
Cold peripheries	4 (3.5%)	7 (3.3%)	6 (2.3%)	18 (3.2%)	35 (3.1%)	0.89	478
Urinary output < 2 mL/kg/hr	1 (1.7%)	4 (3.0%)	5 (2.4%)	9 (1.8%)	19 (2.1%)	0.84	725
**Comorbidities:**							
Presence of one or more comorbidities	77 (28.2%)	119 (33.6%)	140 (35.2%)	172 (29.3%)	508 (31.5%)	0.12	6
Prematurity	20 (18.0%)	38 (17.8%)	48 (17.8%)	85 (15.6%)	191 (16.8%)	0.81	479
Chronic neurological disease	28 (10.5%)	26 (7.7%)	32 (8.1%)	27 (4.6%)	113 (7.1%)	0.012	36
Diabetes	10 (7.3%)	12 (5.1%)	26 (9.0%)	24 (4.2%)	72 (5.9%)	0.032	388
HIV infection	11 (4.1%)	12 (3.6%)	17 (4.5%)	41 (7.1%)	81 (5.2%)	0.065	61
Hypertension	3 (2.0%)	18 (7.5%)	9 (3.1%)	5 (0.9%)	35 (2.8%)	<0.001	366

	Ancestral	Beta	Delta	Omicron	Total	p-value*	Missing†
	**N=275**	**N=357**	**N=399**	**N=587**	**N=1,618**		**0**
	**number (%)**	**number (%)**	**number (%)**	**number (%)**	**number (%)**		
Chronic lung disease	13 (5.0%)	13 (4.0%)	15 (3.9%)	17 (2.9%)	58 (3.8%)	0.53	77
Tuberculosis	8 (3.1%)	12 (3.6%)	11 (2.8%)	9 (1.5%)	40 (2.6%)	0.23	57
Asthma	6 (2.3%)	8 (2.4%)	10 (2.6%)	21 (3.6%)	45 (2.9%)	0.60	45
Cancer	1 (0.9%)	0 (0.0%)	2 (0.7%)	2 (0.4%)	5 (0.4%)	0.55	423
Immune disorder	3 (1.1%)	4 (1.2%)	12 (3.1%)	6 (1.0%)	25 (1.6%)	0.063	43
Chronic kidney disease	7 (2.7%)	12 (3.6%)	8 (2.0%)	2 (0.3%)	29 (1.8%)	0.003	45
**Chronic liver disease**	1 (0.4%)	3 (0.9%)	2 (0.5%)	4 (0.7%)	10 (0.6%)	0.86	47
Hematological disorder	2 (0.8%)	7 (2.1%)	10 (2.6%)	2 (0.3%)	21 (1.3%)	0.011	50
Inflammatory / rheumatological condition	2 (0.8%)	2 (0.6%)	5 (1.3%)	0 (0.0%)	9 (0.6%)	0.073	43

* test for trend across all periods †Calculation of % excludes missing values

Supplementary Table S7: Symptoms, signs and comorbidities of hospitalized children with primary Covid-19 by disease severity

Characteristic	Severe primary Covid-19 N=1121 (69.3%)	Non-severe primary Covid-19 N=497 (30.7%)	Total N=1618 (100%)	p-value	Missing
	Number (%)	Number (%)	Number (%)		
**Respiratory symptoms as reported by parent/caregiver**					
Difficulty or fast breathing	714 (63.9)	65 (13.3)	779 (48.5)	<0.001	12
Cough	599 (55.6)	143 (32.1)	742 (48.7)	<0.001	95
Sore throat	57 (6.5)	21 (5.3)	78 (6.1)	0.41	348
Tachypnoea	524 (48.0)	55 (11.8)	579 (37.2)	<0.001	62
Difficulty breathing	602 (54.1)	15 (3.1)	617 (38.6)	<0.001	20
Rhinorrhoea	172 (21.2)	30 (8.7)	202 (17.5)	<0.001	464
Wheezing	195 (17.8)	8 (1.7)	203 (12.9)	<0.001	42
**Gastro-intestinal symptoms as reported by parent/caregiver**					
Abdominal pain	97 (9.3)	48 (11.0)	145 (9.8)	0.31	142
Diarrhoea	247 (22.7)	126 (28.1)	373 (24.3)	0.024	81
Vomiting	330 (30.2)	153 (34.0)	483 (31.3)	0.15	76
Unable to drink	72 (8.4)	9 (2.6)	81 (6.7)	<0.001	414
**Commonest mucocutaneous symptoms**					
Rash	105 (9.6)	44 (9.8)	149 (9.7)	0.93	79
Erythematous or sticky eyes or bilateral conjunctivitis	32 (3.9)	13 (3.8)	45 (3.9)	0.95	45v2
Erythematous mouth or lips	9 (2.6)	9 (5.4)	18 (3.5)	0.099	1102
Peeling or erythematous fingers, toes, palms, soles or oral /peripheral mucosal inflammation	28 (3.3)	11 (3.2)	39 (3.3)	0.92	432
**Commonest musculoskeletal symptoms**					
Myalgia	44 (4.7)	18 (4.3)	62 (4.6)	0.78	257
Cervical lymphadenopathy	28 (3.3)	4 (1.2)	32 (2.7)	0.037	421
Arthralgia	17 (2.2)	9 (2.7)	26 (2.3)	0.59	501
Inflamed joints	11 (1.4)	5 (1.5)	16 (1.4)	0.90	473
**Commonest neurological symptoms**					
Irritability	185 (17.2)	57 (12.6)	242 (15.9)	0.026	92
Fatigue/malaise	200 (19.0)	35 (7.8)	235 (15.7)	<0.001	120
Floppiness	72 (8.2)	14 (4.0)	86 (7.0)	0.010	393
Headache	50 (5.7)	30 (7.4)	80 (6.2)	0.25	334

Characteristic	Severe primary Covid-19 N=1121 (69.3%)	Non-severe primary Covid-19 N=497 (30.7%)	Total N=1618 (100%)	p-value	Missing
Seizures	30 (8.5)	19 (11.4)	49 (9.5)	0.30	1100
Stiff neck	14 (1.6)	4 (1.1)	18 (1.5)	0.54	407
Photophobia	11 (1.4)	3 (0.9)	14 (1.2)	0.52	468
Ageusia	3 (0.9)	3 (2.0)	6 (1.3)	0.32	1126
Paralysis	11 (1.3)	2 (0.6)	13 (1.1)	0.29	397
Hyposmia / Anosmia	6 (0.8)	2 (0.6)	8 (0.7)	0.66	465
**Other:**					
Fever	553 (50.9)	207 (46.2)	760 (49.5)	0.096	83
Haemorrhage	11 (1.3)	3 (0.9)	14 (1.2)	0.57	406
**Signs on admission**	Number (%)	Number (%)	Number (%)		
Hypotension	124 (12.6)	2 (0.6)	126 (9.4)	<0.001	283
**Respiratory rate (median beats per minute (IQR)**				<0.001	
0-28 days	52 (42-65)	42 (36-48)	45 (40-56)		28
29-365 days	44 (37-56)	38 (30-42)	42 (36-52)		54
1-5 years	36 (30-45)	30 (26-34)	34 (28-40)		73
>5-12 years	28 (24-38)	24 (20-28)	26 (22-35)		43
>12 years	24 (20-30)	20 (18-20)	22 (20-28)		18
Oxygen Saturation (Percent and IQR)	95 (92-98)	98 (96-99)	96 (93-98)	<0.001	211
Tachycardia	487 (44.5)	114 (24.4)	601 (38.5)	<0.001	57
Prolonged capillary refill time	41 (5.1)	8 (2.4)	49 (4.3)	0.044	480
Pale/mottled skin	66 (6.9)	12 (2.9)	78 (5.7)	0.004	252
Chest pain	39 (4.5)	3 (0.7)	42 (3.3)	<0.001	337
Cold peripheries	39 (4.5)	3 (0.7)	42 (3.3)	0.002	478
Urinary output < 2 mL/kg/hr	18 (3.0)	1 (0.4)	19 (2.1)	0.012	725
**Comorbidities**					
At least 1 comorbidity	380 (44.9)	114 (33.6)	494 (41.7)	<0.001	432
Prematurity	158 (19.4)	33 (10.2)	191 (16.8)	<0.001	479
Chronic neurological disease	85 (7.8)	28 (5.7)	113 (7.1)	0.14	36
HIV	61 (5.7)	20 (4.1)	81 (5.2)	0.21	61
Diabetes mellitus	47 (5.4)	25 (7.1)	72 (5.9)	0.24	388
Heart problem	67 (6.2)	13 (2.7)	80 (5.1)	0.004	44
Chronic lung disease	48 (4.5)	10 (2.1)	58 (3.8)	0.021	77
Asthma	35 (3.2)	10 (2.1)	45 (2.9)	0.20	45

Characteristic	Severe primary Covid-19 N=1121 (69.3%)	Non-severe primary Covid-19 N=497 (30.7%)	Total N=1618 (100%)	p-value	Missing
Tuberculosis (TB)	33 (3.1)	7 (1.5)	40 (2.6)	0.065	57
Hypertension	28 (3.1)	7 (2.0)	35 (2.8)	0.27	366
Chronic kidney disease	23 (2.1)	6 (1.2)	29 (1.8)	0.22	45
Immune disorder	22 (2.0)	3 (0.6)	25 (1.6)	0.038	43
Haematological disorder	16 (1.5)	5 (1.0)	21 (1.3)	0.47	50
Respiratory disease other than TB	11 (2.3)	1 (0.5)	12 (1.7)	0.086	911
Chronic liver disease	6 (0.6)	4 (0.8)	10 (0.6)	0.54	47
Inflammatory / rheumatological condition	7 (0.6)	2 (0.4)	9 (0.6)	0.57	43
Cancer	4 (0.5)	1 (0.3)	5 (0.4)	0.67	423

Supplementary Table S8: Description of most abnormal laboratory value* during the admission of children with primary Covid-19, by disease severity

Most abnormal laboratory value from medical record	Primary Covid-19 (severe +non-severe)	Severe PrimaryCovid-19 n=1121		Non-severe primary Covid-19 N=497		p-value	Missing / not done
	**measurement**	N*	measurement	N*	measurement		
Temperature (Celsius)	37.0 (36.5-37.5)	1025	37.0 (36.6-37.6)	380	36.8 (36.5-37.5)	<0.001	213
Total white blood cell (WBC) count (x10^9/L)	11.3 (7.9-16.7)	771	11.4 (7.9-16.9)	253	11.1 (7.8-15.6)	0.28	594
Neutrophils	6.0 (2.8-11.7)	682	6.1 (3.0-11.6)	216	5.8 (2.4-13.2)	0.53	720
Lymphocytes (x10^9/L)	4.0 (2.1-7.4)	669	3.8 (1.9-7.0)	209	4.8 (2.6-8.2)	0.002	740
Platelets (x10^9/L)	362.5 (258.0-480.0)	778	365.0 (244.0-486.0)	258	355.0 (274.0-465.0)	0.96	582
Creatinine (umol/L)	37.0 (26.0-51.0)	699	37.0 (25.0-53.0)	249	38.0 (28.0-50.0)	0.41	670
Sodium (mmol/L)	137.0 (134.0-141.0)	725	137.0 (134.0-141.0)	245	136.0 (134.0-139.0)	0.050	648
Potassium (mmol/L)	4.8 (4.0-5.4)	713	4.8 (4.0-5.5)	240	4.5 (4.1-5.2)	0.037	665
Urea (BUN) (mmol/L)	4.2 (2.9-6.7)	534	4.3 (2.9-7.0)	181	3.9 (2.9-5.7)	0.13	903
CRP (mg/L)	16.0 (4.0-59.0)	675	18.0 (4.0-68.0)	217	11.0 (2.0-40.0)	0.002	726
INR	1.1 (1.0-1.2)	183	1.1 (1.0-1.2)	72	1.1 (1.0-1.2)	0.30	1363
D-dimer (ng/L)	1.8 (0.6-6.9)	184	2.1 (0.8-7.5)	61	0.7 (0.3-3.1)	<0.001	1373
PT (seconds)	12.4 (11.4-14.0)	151	12.4 (11.4-14.5)	59	12.5 (11.4-13.4)	0.93	1408
APTT/APTR	25.3 (21.0-31.4)	147	25.8 (21.0-33.0)	55	23.9 (20.9-30.0)	0.54	1416
Creatine kinase (U/L)	87.0 (39.5-204.5)	125	99.0 (45.0-235.0)	43	64.0 (35.0-170.0)	0.056	1450
Glucose (mmol/L)	6.1 (4.9-7.9)	180	6.2 (5.0-8.6)	43	5.5 (4.7-6.7)	0.14	1395
Fibrinogen (mg/dl)	3.2 (2.0-4.9)	120	3.1 (1.9-5.0)	43	3.3 (2.1-4.5)	0.75	1455
Procalcitonin (ng/mL)	1.0 (0.1-8.8)	144	1.4 (0.2-12.7)	43	0.4 (0.1-3.9)	0.091	1431
Troponin (ng/Ml)	10.0 (10.0-39.5)	140	11.0 (10.0-48.0)	36	10.0 (10.0-10.0)	0.029	1442
IL-6 (pg/Ml)	5.8 (4.6-48.7)	3	5.8 (4.6-48.7)	0	Not done	N/A†	1615
Pro-BNP (pg/Ml)	144.0 (29.0-1270.0)	40	207.0 (32.5-1824.0)	5	29.0 (5.8-52.0)	0.080	1573

*Numbers do not add up to 1618 in all rows due to investigations not indicated / not done / missing information; rounded to one decimal place †not applicable

Supplementary Table S9: Factors associated with severe disease

	Univariable		Multivariable	
**Characteristic**	**Risk ratio**	**p-value**	**Adjusted Risk ratio**	**p-value**
**Wave**				
Ancestral	Reference		Reference	
Beta	0.98 (0.89 – 1.08)	0.754	0.96 (0.86 – 1.06)	0.423
Delta	0.91 (0.82 1.01)	0.064	0.89 (0.81 – 0.99)	0.026
Omicron	0.96 (0.87 – 1.05)	0.330	0.92 (0.84 – 1.01)	0.075
**Nutritional status**				
Normal	Reference		Reference	
Underweight	1.15 (1.08 – 1.23)	<0.001	1.09 (1.02 – 1.17)	0.013
Overweight	0.96 (0.83 – 1.12)	0.596	0.94 (0.81 – 1.09)	0.393
**Age category**				
0-28 days	Reference		Reference	
29-365 days	1.48 (1.28 – 1.71)	<0.001	1.49 (1.30 – 1.72)	<0.001
1-5 yrs	1.24 (1.07 – 1.44)	0.006	1.26 (1.09 – 1.46)	0.002
5-12 yrs	1.13 (0.97 – 1.33)	0.118	1.13 (0.97 – 1.31)	0.128
>12 yrs	1.53 (1.3 – 1.78)	<0.001	1.51 (1.30 – 1.77)	<0.001
**Comorbidities**				
none	Reference		Reference	
1 or more	0.86 (0.81 – 0.91)	<0.001	1.14 (1.07 – 1.22)	<0.001

Supplementary Material: Standard operating procedure for identifying incidentals versus primary COVID-19

The following questions and variables will be used to identify reason for hospitalization:

Question	Variable
2.2.a.1 Specify new illness	specify_new_illness
2.3.a Specify multisystem inflammatory syndrome	specify_multisystem_inflam

1. **Primary COVID diagnosis:** Children who had a positive SARS-CoV-2 PCR or antigen and any of the following symptoms
   - Abdominal pain
   - Vomiting
   - Respiratory illness
   - Pneumonia
   - Diarrhea
   - MIS-C as defined above

2. **Incidental COVID-19 diagnosis** – none of the above symptoms but positive SARS-CoV-2 PCR or antigen test with any of the following:
   - Burn wounds
   - Accidental: Gunshot wound, accident, head injury
   - Fracture
   - Surgical procedure
   - Audiology screening
   - Organophosphate poisoning / toxin ingestion
   - Parasuicide

3. **<u>For possible discussion, though please try and review each case and indicate your choice and motivation for your choice:</u>**
    - Seizures / severe neurological manifestations with positive antibodies or PCR
    - Chronic illness: Diabetes, heart disease, cancer, kidney

1. Statisticians will group participants according to the above criteria.

2. Adjudication group to make final decision

During data analysis, an expert group of nine paediatricians adjudicated all cases to determine if they were primary or incidental Covid-19. Cases difficult to classify were re-reviewed independently by two different paediatricians, blinded to site, guided by the SOP. Discordant classifications were adjudicated by a third paediatrician blinded to the previous classifications. Furthermore the diagnoses of children with incidental Covid-19 were reviewed and the charts of children with any diagnoses that flagged primary Covid-19 e.g. respiratory illness were re-reviewed. Any remaining discrepancies were discussed during consensus meetings. Despite these discussions, a subset of cases were classified as difficult to classify.
